# The landscape of chromatin accessibility in skeletal muscle during embryonic development in pigs

**DOI:** 10.1186/s40104-021-00577-z

**Published:** 2021-05-03

**Authors:** Jingwei Yue, Xinhua Hou, Xin Liu, Ligang Wang, Hongmei Gao, Fuping Zhao, Lijun Shi, Liangyu Shi, Hua Yan, Tianyu Deng, Jianfei Gong, Lixian Wang, Longchao Zhang

**Affiliations:** Key Laboratory of Animal (Poultry) Genetics Breeding and Reproduction, Ministry of Agriculture; Institute of Animal Science, Chinese Academy of Agricultural Sciences, Beijing, 100193 China

**Keywords:** Chromatin accessibility, Embryo, Pig, Skeletal muscle, Transcriptome

## Abstract

**Background:**

The development of skeletal muscle in pigs during the embryonic stage is precisely regulated by transcriptional mechanisms, which depend on chromatin accessibility. However, how chromatin accessibility plays a regulatory role during embryonic skeletal muscle development in pigs has not been reported. To gain insight into the landscape of chromatin accessibility and the associated genome-wide transcriptome during embryonic muscle development, we performed ATAC-seq and RNA-seq analyses of skeletal muscle from pig embryos at 45, 70 and 100 days post coitus (dpc).

**Results:**

In total, 21,638, 35,447 and 60,181 unique regions (or peaks) were found across the embryos at 45 dpc (LW45), 70 dpc (LW70) and 100 dpc (LW100), respectively. More than 91% of the peaks were annotated within − 1 kb to 100 bp of transcription start sites (TSSs). First, widespread increases in specific accessible chromatin regions (ACRs) from embryos at 45 to 100 dpc suggested that the regulatory mechanisms became increasingly complicated during embryonic development. Second, the findings from integrated ATAC-seq and RNA-seq analyses showed that not only the numbers but also the intensities of ACRs could control the expression of associated genes. Moreover, the motif screening of stage-specific ACRs revealed some transcription factors that regulate muscle development-related genes, such as MyoG, Mef2c, and Mef2d. Several potential transcriptional repressors, including E2F6, OTX2 and CTCF, were identified among the genes that exhibited different regulation trends between the ATAC-seq and RNA-seq data.

**Conclusions:**

This work indicates that chromatin accessibility plays an important regulatory role in the embryonic muscle development of pigs and regulates the temporal and spatial expression patterns of key genes in muscle development by influencing the binding of transcription factors. Our results contribute to a better understanding of the regulatory dynamics of genes involved in pig embryonic skeletal muscle development.

**Supplementary Information:**

The online version contains supplementary material available at 10.1186/s40104-021-00577-z.

## Background

The growth of skeletal muscle has received considerable attention because its dysfunction can cause debilitating musculoskeletal disorders [1]. Previous research has shown that the development of skeletal muscle is a complex process that includes the formation of embryonic muscle fibers, the expansion of postnatal muscle fibers, and the regeneration of adult muscles [2]. The number of muscle fibers is essentially fixed during the embryonic period [3]. Additionally, postnatal fiber hypertrophy depends on the total number of muscle fibers within a muscle [4]. Therefore, the embryonic muscle development process is extremely important. Research on the genetic mechanisms affecting muscle development, particularly during embryonic stages, will be beneficial for improving pork production methods and for expanding pig breeding strategies. Moreover, pigs are more closely related to humans in terms of their size, anatomy, genome, and physiology than other non-primate species (e.g., traditional rodent models); thus, pigs are more suitable than other species for research on human health [5, 6].

Muscle development in pig embryos takes place in two growth waves: the first occurs during days 35–60 of the embryonic stage and involves the formation of primary fibers, and the second occurs during days 54–90 of the embryonic period and mostly results in the formation of secondary fibers [7]. The primary fibers, which serve as the initial muscle fibers, are formed by cell fusion, and myoblasts then attach to the surface of primary fibers and fuse to form secondary fibers. The morphology of primary fibers and secondary fibers can be easily recognized even before prenatal day 80. Primary fibers have a tubular appearance, and their center consists of a nucleus or myofibril-free region. In contrast, secondary fibers generally surround the primary fibers and exhibit a solid appearance. In addition, the volume of primary fibers is 2–3 times that of secondary fibers during most of the prenatal period [3]. This process is regulated by multiple mechanisms at the epigenetic, transcriptional, and posttranscriptional levels [2, 8]. As one of the most common types of regulatory factors, transcription factors (TFs), which can bind to target DNA sequences via their DNA-binding domains to promote or inhibit mRNA transcription play crucial roles in skeletal muscle development [9–11]. In addition, studies have shown that some TFs called pioneer factors can establish chromatin accessibility by replacing or binding nucleosome DNA or by recruiting chromatin-remodeling agents [12, 13]. Quantitative trait locus (QTL) analysis and 3D genome assembly have shown that changes in chromatin accessibility ultimately alter the long-range effects of TFs [14]. Therefore, studying the interaction mechanism between TFs and chromatin accessibility during muscle development is particularly important.

Chromatin accessibility, an important component of epigenomics, can directly reflect the effects of chromatin structural modification on gene transcription. In eukaryotic lineages, the binding of TFs results in transcriptional activation, which is closely related to the disruption of nucleosome assembly at promoters, enhancers, insulators, and locus control regions. Thus, regulatory DNA affects the openness or accessibility of a genomic locus of remodeled chromatin [15]. Several methods have been used to profile chromatin accessibility, and those include deoxyribonuclease I (DNase I)-hypersensitive site sequencing (DNase-seq), assay for transposase-accessible chromatin with high-throughput sequencing (ATAC-seq), and formaldehyde-assisted isolation of regulatory elements sequencing (FAIRE-seq) [15]. Among these, ATAC-seq is the preferred method due to its strong advantages: it requires a small input of cells, has a shorter sample-processing period than the other techniques, and has been applied in a variety of studies [16–18]. Although several studies have recently performed ATAC-seq for the analysis of numerous tissues, such as the liver, frontal cortex, lung and *longissimus dorsi* muscle, the landscape of chromatin accessibility in skeletal muscle during embryonic stages remains poorly elucidated [19, 20].

In this study, to investigate the dynamics of chromatin accessibility during muscle development in pig embryos, we used ATAC-seq and RNA-seq to analyze the chromatin accessibility and transcriptome of *longissimus dorsi* tissue of Large White (LW) pigs at different embryonic stages [45, 70, and 100 days post coitus (dpc)]; denoted LW45, LW70, and LW100, respectively. The results of this work will provide a theoretical basis for comparing the molecular mechanisms of embryonic muscle development among different stages, which will broaden our knowledge of epigenetics during muscle development.

## Methods and materials

### Ethics statement

All experiments on pigs were performed under the guidance of the Chinese Academy of Sciences and the Institute of Animal Science, Chinese Academy of Agricultural Sciences (CAAS), China.

### Sample description

**Fig. 1:**
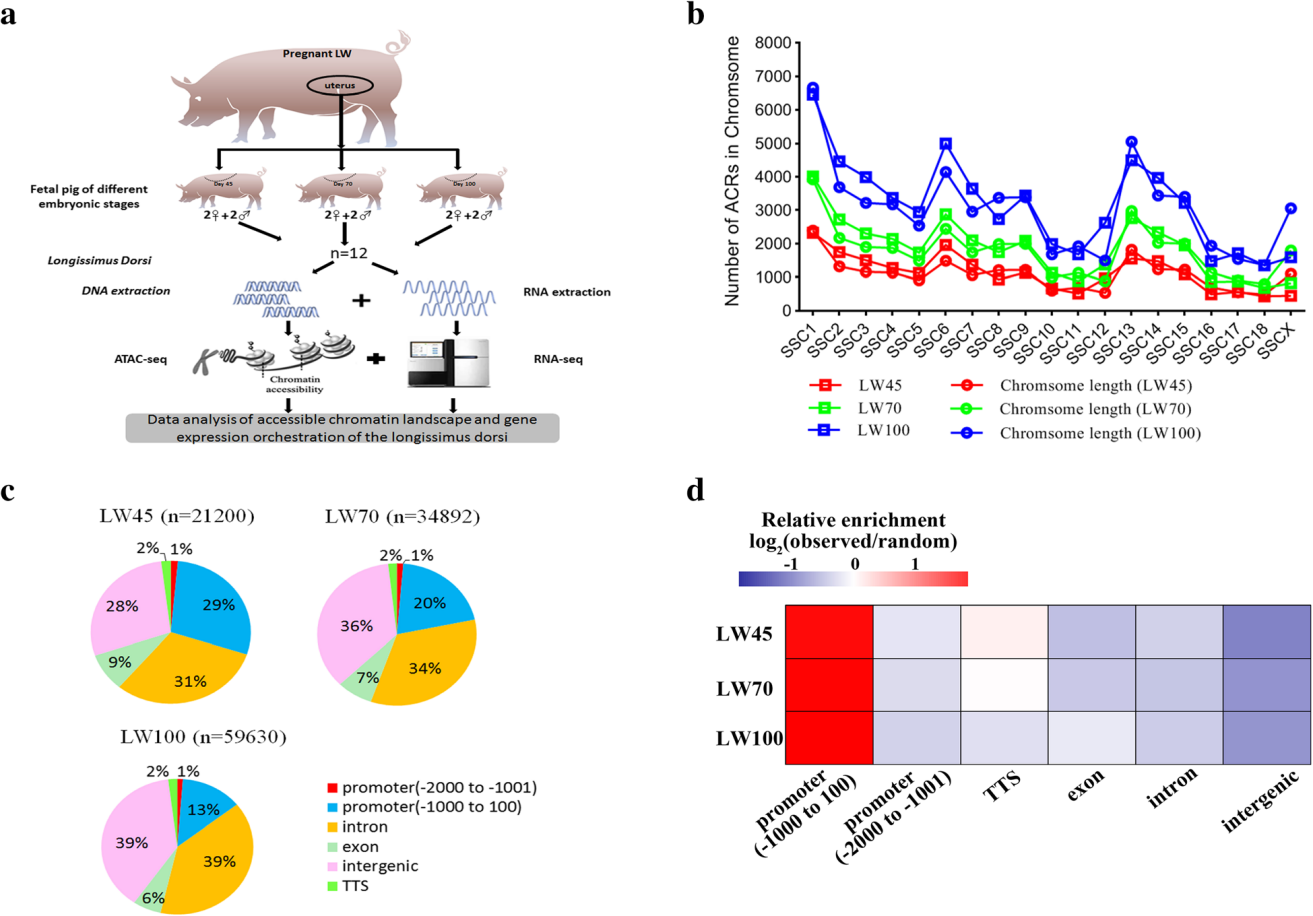
Overview of the ATAC-seq libraries. **a** Schematic representation of the experiment. **b** Distributions of ACR numbers across different chromosomes. **c** Percentages of ACRs in different genomic regions. **d** Fold enrichment of ACRs in different genomic regions

All LW purebred pigs used in this study were obtained from an experimental pig farm at the Institute of Animal Science, CAAS (Beijing, China). Three full-sib sows were slaughtered at 45, 70 and 100 dpc, which approximately corresponded to the primary fiber establishment stage, the secondary fiber development stage and the total number of fibers fixed stage, respectively. At each stage, four full-sib embryos (two males and two females) were selected, and fresh *longissimus dorsi* muscle tissue was isolated between the 5th and 6th ribs of each embryo (Fig. 1a). The tissue was immediately placed in liquid nitrogen for storage.

### ATAC-seq and data analysis

A total of 12 samples were used to construct libraries for ATAC-seq. First, approximately 50,000 fresh cells were collected from each sample. After several centrifugations, 50 μL of tagmentation reaction mix (25 μL of 2× reaction buffer from a Nextera kit, 2.5 μL of Nextera Tn5 transposase from the Nextera kit, and 22.5 μL of nuclease-free H_2_O) was added to each sample, and the reactions were immediately incubated for 30 min at 37 °C and then subjected to eight cycles of PCR amplification. A MinElute PCR purification kit (Qiagen) was used to purify the libraries, and an Agilent Bioanalyzer 2000 was used to assess the quality of the libraries. The ATAC-seq DNA was sequenced in the 150 bp paired-end sequencing mode with a NovaSeq 6000 platform.

The raw fragments were trimmed with Trimmomatic to eliminate reads containing sequence adapters as well as low-quality base pairs [21]. After trimming, the quality of the fragments was evaluated with FastQC. The reads were aligned to the swine reference genome (*Sus scrofa* 11.1.94) using BWA-MEM with the parameter –v 3 [22]. Duplicated fragments, fragments with a mapping quality of less than 30, fragments mapped to the Y chromosome and mitochondrial DNA were removed with SAMtools [23]. The accessible chromatin regions (ACRs) in each individual were called by MACS 2.0 with the parameter -f BAMPE -q 0.01 [24]. To further verify the most representative accessible regions of the genome among the samples, the ACRs that were shared by all samples at the same embryonic age were merged using BEDTools [25]. DeepTools was used to convert the BAM files to BigWig files for visualization of the genome-wide peaks in Integrative Genomics Viewer as well as for investigation of the signal distribution in and around the gene bodies [26]. The HOMER annotatePeaks function was used for peak annotation using *Sus scrofa* 11.1.94 [27]. The ATAC-seq peaks that were within − 2 kb to + 100 bp of transcription start sites (TSSs) were selected as promoter peaks. To evaluate the enrichment of peaks in different genomic regions (observed region/random region), we first used BEDtools to extract random positions on random chromosomes [25] and these random regions were then annotated in the genome using a custom script. This process was repeated 5000 times, and the average value of the annotation result for different genomic regions was regarded as a random region. To investigate the relationship between the number of peaks and the chromosome length, we first normalized the length of the chromosome (normalized chromatin length = total peak number*chromatin length/total chromatin length), and then calculated the Pearson correlation coefficient between the normalized chromatin length and the number of peaks. The specific and common peaks among different embryonic ages were identified using BEDTools [25]. To investigate the connection between the peak length and the gene expression level, the genes that contained a single ACR in the proximal promoter region were sorted according to the ACR length and were then divided into three equal groups, namely, the top, middle and bottom groups. If several ACRs had the same length and could not be easily assigned to a group, it was assigned to the groups that included the greatest numbers of peaks of similar length (i.e., if 90 ACRs are divided into three groups according their length, each group should theoretically contain 30 ACRs; however, if the length of the 29th and 30th ACRs of the bottom group is equal to that of the first ACR of the middle group, the first peak in the middle group will be assigned to the bottom group). For genes containing multiple ACRs (> 1 ACR) in the proximal promoter region, we first calculated the maximum length and the total length of ACRs in the proximal promoter of each gene and then used the above method to obtain the three groups. DEseq2 was used to analyze the differential peak intensity (DPI) among different embryonic stages [28]. The following thresholds were used to define significant DPI: adjusted *P*-value< 0.05 and a log_2_|fold change (FC)| > 1. Gene Ontology (GO) and Kyoto Encyclopedia of Genes and Genomes (KEGG) pathway analyses were performed using the R package clusterProfiler [29]. A *p*-value of 0.01 was used as the significance cutoff for GO term and pathway identification. To investigate the cis-regulation elements (CREs) in genomic regions, a 200-bp region from − 100 to + 100 relative to the peak center was screened for motifs using the HOMER findMotifsGenome function with the default parameters [27]. The significance cutoff for motif identification was a *P*-value of 0.01.

### RNA-seq library preparation, sequencing and data processing

RNA was extracted from all the samples using a TruSeq Stranded Total RNA Ribo-Zero H/M/R Kit (Illumina RS-122-2201) and then subjected to quality assessment using 1.5% agarose gel electrophoresis (to verify integrity) and a NanoDrop instrument (to estimate the purity and concentration). High-quality RNA samples were used to prepare the RNA-seq libraries, and these were then sequenced as paired-end 150 bp sequences with a HiSeq X platform.

For all the samples, the raw reads were first trimmed using Trimmomatic to remove adapters and low-quality base pairs [21]. The trimmed reads were then aligned to the reference genome (*Sus scrofa* 11.1.94) using STAR [30]. The alignment results were processed with SAMtools to remove unmapped reads [23]. The read counts within exons were calculated using HTSeq [31]. The methods used to determine the differentially expressed genes (DEGs) and the significance threshold were the same as those described for ATAC-seq. The fragments per kilobase per million mapped reads (FPKM) values were determined to measure the gene expression levels using Cufflinks; this method is one of the most common methods at present [32]. All downstream analyses were based on the genes with FPKM values greater than 1 in at least three samples at one of the embryonic stages.

## Results

### Genome-wide identification of ACRs during pig embryonic development

To examine the genome-wide ACRs involved in muscle development, we profiled the accessibility of chromatin at 45, 70 and 100 days during pig embryonic development by ATAC-seq. ATAC-seq was performed with 12 samples, and a total of 152–170 million reads were uniquely mapped to the reference genome (Additional file 1: Table S1). We first assessed the quality of the libraries based on the peak signal distributions and the lengths of the inserts. Detailed information on the high-quality libraries derived from each individual can be found in Additional file 2: Fig. S1 and Additional file 2: Fig. S2, which show that all the libraries exhibited the expected fragment length, contained abundant nucleosome-free and mononucleosomal spanning fragments (Additional file 2: Fig. S1), and exhibited the highest peak signal across the whole gene body in the TSS (Additional file 2: Fig. S2). Numerous peaks with high confidence were obtained based on the 12 libraries (Additional file 1: Table S1). As shown in Table S1, more peaks were acquired from the samples of older embryos, which indicated that chromatin is globally more accessible in older embryos than in younger embryos. To further verify the most representative accessible regions of the genome among samples, we merged the peaks that were shared among all four samples in the same group. Ultimately, 21,638, 35,447 and 60,181 unique regions (or peaks) with 0.50%, 0.89% and 1.89% coverage of the swine genome were found at 45, 70 and 100 dpc, respectively. All downstream analyses were based on these regions.

We investigated the relationship between the ACR numbers and chromosome length and found that chromatin with longer chromosomes displayed a higher density of peaks (*r*^*2*^ = 0.84 (LW45), *r*^*2*^ = 0.90 (LW70), *r*^*2*^ = 0.89 (LW100)) (Fig. 1b). To further profile the ACR distribution across the whole genome, we evaluated the relative positions of the peaks in the genome (Fig. 1c). At each stage, numerous peaks were annotated in intron and intergenic regions, and these accounted for approximately 2/3 of all peaks. Approximately 30%, 21%, and 14% of the peaks were identified in promoter regions at 45, 70 and 100 dpc, respectively. Among the peaks located in promoter regions, more than 91% were annotated within − 1 kb to 100 bp of the TSS. Few peaks were identified in transcriptional termination sites (TTSs) and exonic regions. In addition, the percentage of peaks located in proximal promoter regions (− 1 kb to 100 bp away from the TSS) gradually decreased as the embryo developed, whereas the percentages of peaks within intron and intergenic regions increased during development. At all the stages, the ACRs were relatively enriched in promoter and exon regions, particular in proximal promoter regions, instead of intergenic regions (Fig. 1d).

**Fig. 2:**
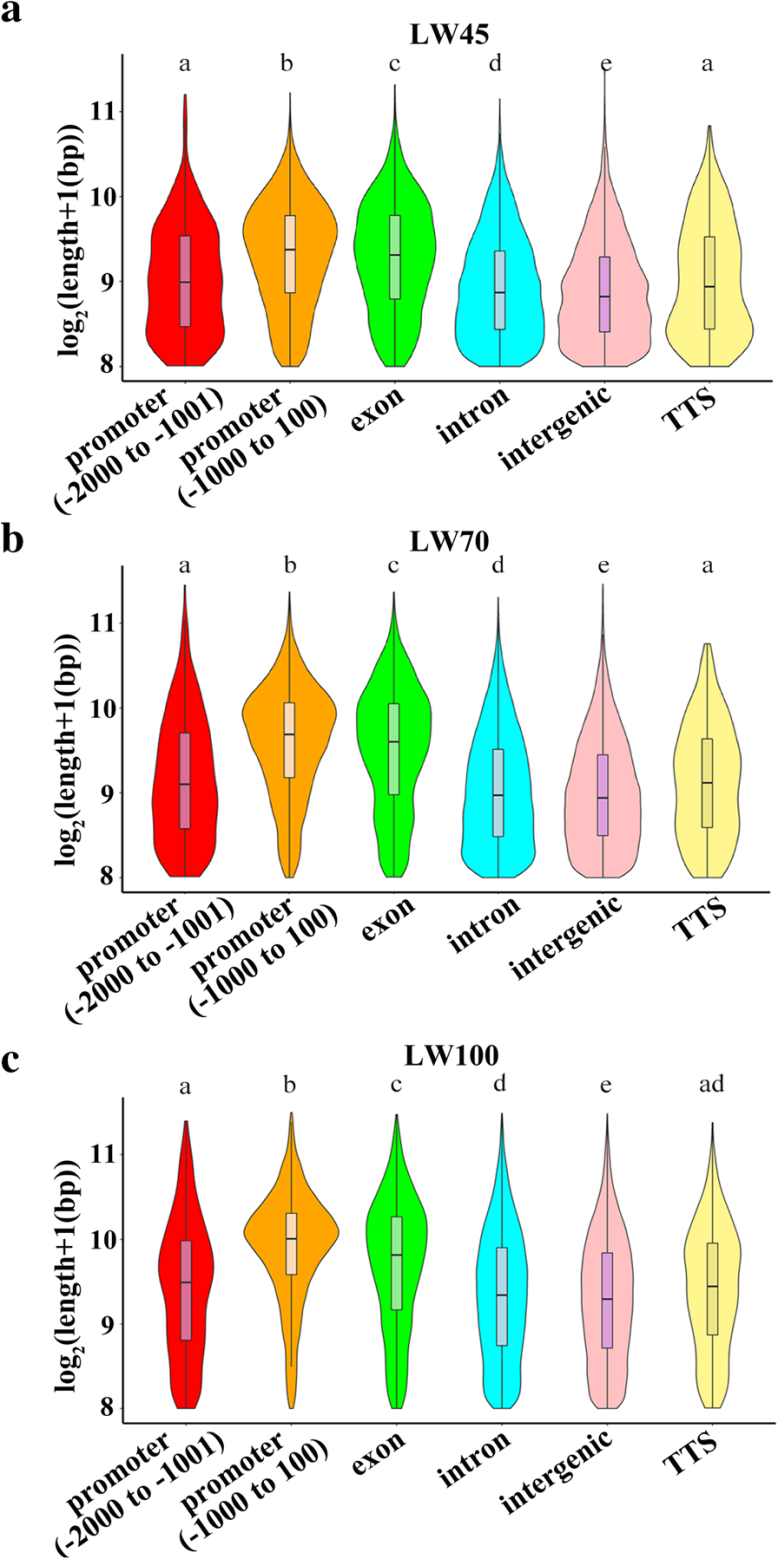
Overview of the ACR lengths in different genomic regions. **a-c**: Distributions of ACR lengths in different genomic regions at the LW45, LW70 and LW100 stages. Different letters above the violins indicate significant differences between the groups based on Tukey’s honestly significant difference test (*P* < 0.05)

We further investigated the average lengths of peaks in different genomic regions (Fig. 2a-c) and found that peaks within proximal promoter regions were longer than those in other regions, whereas the shortest peaks were found in intergenic regions.

### Coordination of the regulation of gene expression by ACRs

**Fig. 3:**
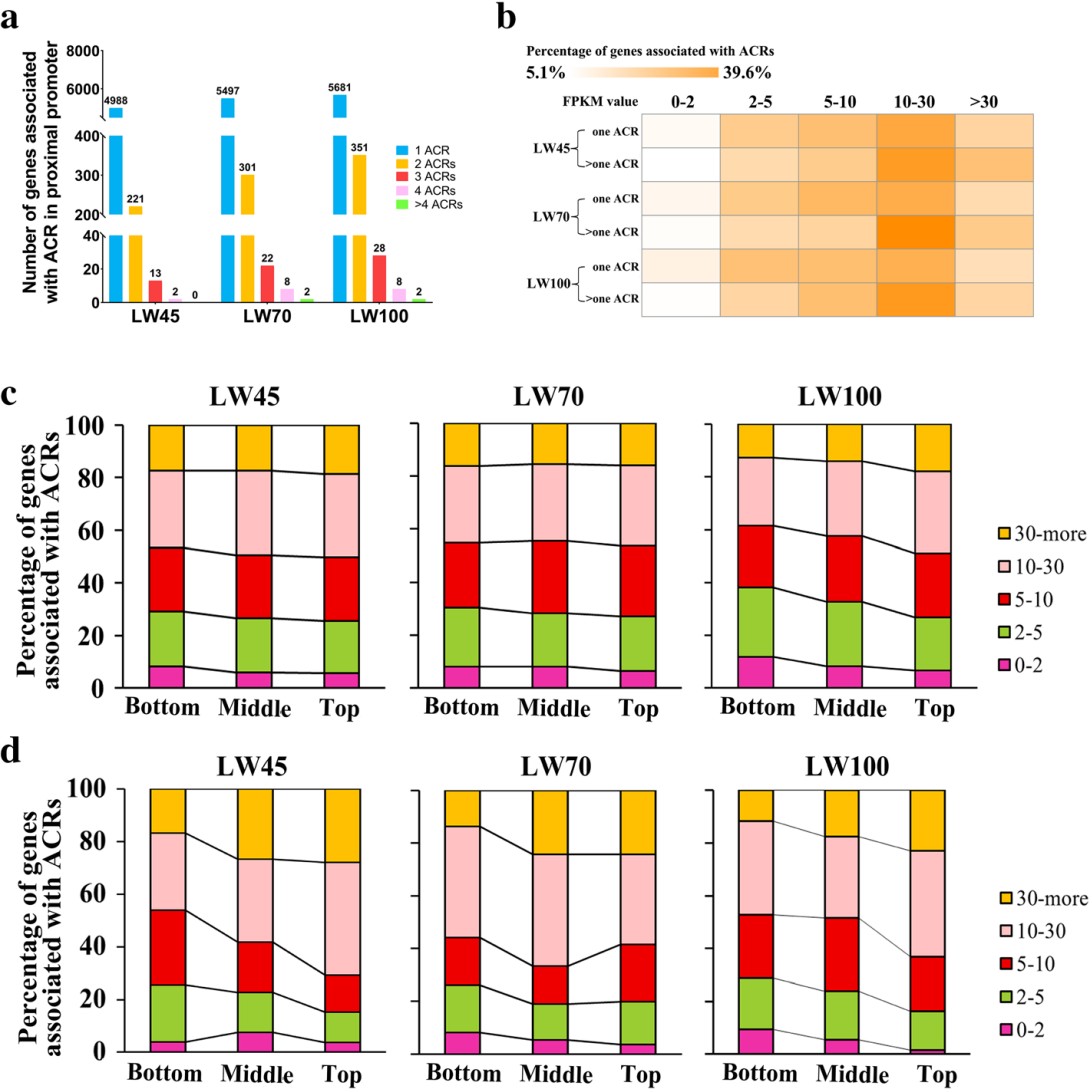
Expression levels of genes associated with ACR numbers.**a** Statistics for ACR numbers in the proximal promoter regions of genes. **b** Heatmap of the percentages of ACR-associated gene expression levels in different groups. The terms “One ACR” and “> one ACR” correspond to the groups of genes that contained one ACR and those that contained multiple ACRs in their proximal promoter regions, respectively. The expression levels of the genes were divided into five groups based on the FPKM values: 0–2, 2–5, 5–10, 10–30, and > 30. **c-d** Percentages of ACR-associated gene expression levels in different groups. The genes were grouped according to whether they contained a single ACR (Fig. 3c) or multiple ACRs (Fig. 3d) in their proximal promoters according to the longest peak length and were then divided into three equal groups (the top, middle and bottom groups). The expression levels of the genes were divided into five groups based on the FPKM values: 0–2, 2–5, 5–10, 10–30, and 30-more

RNA-seq was conducted using the same individuals as ATAC-seq to explore the effect of ACRs on gene expression. The basic information of each RNA library is listed in Additional file 3: Table S2. The results from a cluster analysis showed that the four replicates of each stage were all grouped together, which indicated that our experiment exhibits good repeatability (Additional file 2: Fig. S3). To explore the regulatory role of chromatin accessibility in gene expression, we performed further analysis of ACRs annotated to proximal promoter regions because the ACRs were extremely enriched in these regions, as shown in Fig. 1d. First, we found that more than 90% of the genes annotated by those ACRs contained only one ACR in their proximal promoter region (Fig. 3a). We then analyzed the relationship between the ACR number in the proximal promoter and the gene expression level. At all stages, the proportion of genes with high expression (FPKM> 30) in the group with more than one ACR was greater than that in the group with one-ACR (Fig. 3b). Moreover, we divided the ACRs into three groups, namely, the top, middle and bottom groups, according to the ACR length to investigate the connection between the ACR length and the gene expression level. The results showed that the proportion of highly expressed genes (FPKM > 30) gradually decreased from the top group to the bottom group at most stages regardless of whether the genes had one or multiple ACRs in the proximal promoter region. At all stages with the exception LW45 stage, the proportion of low-expressed genes (FPKM < 2) increased as the peak length declined (Fig. 3c-d and Additional file 2: Fig. S4). These results demonstrate that genes with longer ACRs might exhibit comparatively higher expression levels.

### Identification of stage-specific and differential ACRs during embryonic development

**Fig. 4:**
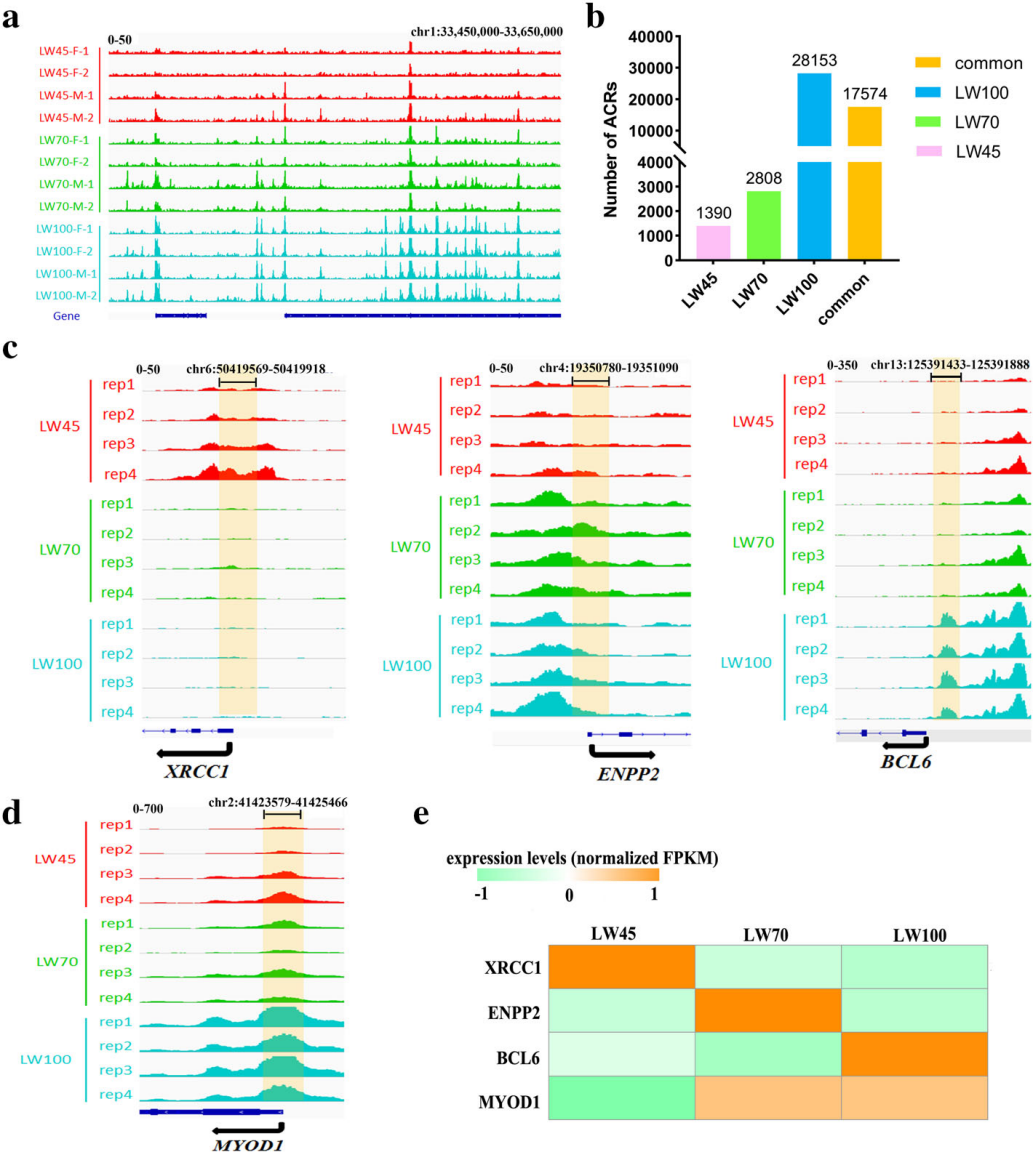
ACR changes during muscle development. **a** Visualization of ACRs at the LW45, LW70 and LW100 stages within a region on Chr1. **b** Statistics for stage-common and stage-specific ACRs at the LW45, LW70 and LW100 stages. **c** Visualization of stage-specific ACRs related genes at different stages. **d** Visualization of stage-common ACRs related genes at different stages. **e** Heatmap of the expression of *XRCC1*, *ENPP2*, *BCL6* and *MYOD1* at the LW45, LW70 and LW100 stages

Although a large number of ACRs were detected at all stages examined in this research, differences were observed among the stages (Fig. 4a) when we compared the genome-wide ACRs obtained from the different samples. In total, 1390, 2808 and 28,153 stage-specific peaks were identified at LW45, LW70 and LW100, respectively (Fig. 4b). For example, *XRCC1*, which can control a temporally responsive DNA repair process to advance the muscle differentiation program, contained an ACR in its proximal promoter region specifically at LW45 (Fig. 4c) [33], and *ENPP2*, which can regulate WNT/β-catenin signaling to control myogenic differentiation, displayed an LW70-specific ACR in the proximal promoter region (Fig. 4c) [34]. In contrast, *BCL6*, which is related to the process of terminal differentiation in muscle cells, showed an LW100-specific ACR (Fig. 4c) [35]. These results demonstrate that ACRs vary dynamically. The changes in ACRs at each stage were consistent with the expression levels of the genes shown in Fig. 4e. The highest expression levels of *XRCC1* and *ENPP2* were observed at the LW45 and LW70 stages, respectively, whereas the highest expression level of *BCL6* was observed at the LW100 stage. We then explored the trends of the changes in the expression levels of stage-specific ACR-related genes at all stages (Additional file 2: Fig. S5a) and the results showed that many of these genes displayed the higher expression levels at their corresponding stages. In total, our results indicated that ACRs counld regulate gene expression partly by altering the binding sites for TFs.

**Fig. 5:**
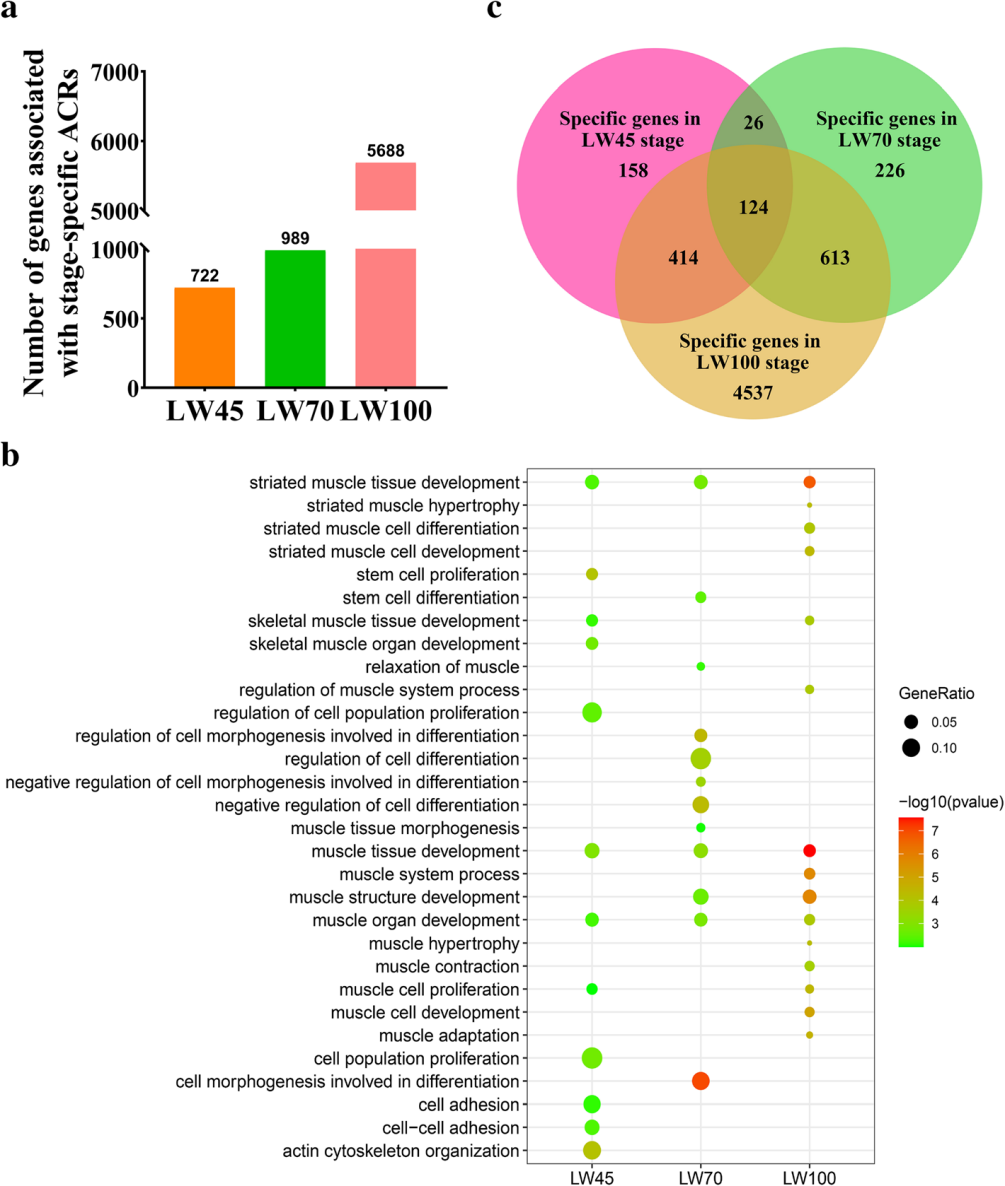
Functional analysis of genes associated with stage-specific ACRs. **a** Numbers of genes related to stage-specific ACRs. **b** Some of muscle related BPs in the LW45, LW70 and LW100 groups. The dot color represents the statistical significance level of enrichment (*P* value); the dot size indicates the proportion of genes enriched for the corresponding terms. **c** Numbers of shared and stage-specific genes associated with specific ACRs at the LW45, LW70 and LW100 stages

We also identified 17,574 common peaks that were shared among all three embryonic stages in this study (Fig. 4b). However, the intensities of some of these peaks differed among the different groups. For example, in the gene body and promoter regions of *MYOD1*, an important regulator that can affect muscle development, chromatin became increasingly accessible from LW45 to LW100, and the peak intensity at the LW100 stage was significantly higher than that at other stages (*P* < 0.05) (Fig. 4d) [36]. Correspondingly, the expression level of *MYOD1* gradually increased during embryonic development (Fig. 4e). We performed DPI analysis of the common peaks among the different embryonic stages (Table 1). The LW70 vs LW45, LW100 vs LW70 and LW100 vs LW45 comparisons showed that 145, 1726 and 2687 ACRs exhibited DPIs, respectively. We then analyzed the correlation between the peak intensity and expression level of each DPI-related gene and found that the correlation coefficient is between 0 and 0.98 (Additional file 2: Fig. S5b). This result suggests that the changes in the intensity of ACRs associated with these genes might also affect their expression. In addition, as shown in Fig. 4b and Table 1, more peaks with significantly different intensities were obtained in the LW100 vs LW70 comparison or in stage-specific ACRs at the LW100 stage, respectively, which suggests that a widespread change in chromatin accessibility occurs during muscle development, primarily during the formation of secondary fibers.

**Table 1:** Results of the DPI analysis of common peaks

Group	Down	Up	Total
LW70 vs. LW45	127	18	145
LW100 vs. LW70	1412	314	1726
LW100 vs. LW45	2224	463	2687

### Distinct regulation of genes at different stages during skeletal muscle development

To further elucidate the regulatory roles of genes with stage-specific ACRs as well as DPIs during embryonic muscle development, we investigated the peak-related genes and their functions. A total of 722, 989, and 5688 genes were found to have stage-specific ACRs at the LW45, LW70 and LW100 stages, respectively (Fig. 5a). GO analyses were performed to assess the biological functions associated with the stage-specific ACR-related genes, and the results showed that genes with stage-specific ACRs showed significant enrich for many different muscle-related biological processes (BPs) (Additional file 4: Table S3 and Fig. 5b). For example, genes associated with the LW45-specific peaks were significantly enriched in the following BPs: actin cytoskeleton organization, muscle tissue development and actin filament organization. Genes with stage-specific ACRs at LW70 were enriched in muscle tissue morphogenesis, muscle structure development and muscle organ development. In addition, the genes identified at LW100 stage were enriched in the following BPs: muscle hypertrophy, striated muscle hypertrophy and muscle tissue development. The KEGG results are displayed in Additional file 4: Table S3. A total of four, nine and 68 pathways were identified at LW45, LW70 and LW100 stages, respectively. The Hippo signaling pathway was enriched in all stages, and the calcium signaling pathway, Wnt signaling pathway and TGF-beta signaling pathway were found at both the LW70 and LW100 stages.

Notably, 124 genes with stage-specific ACRs overlapped among the three stages, which suggested that these genes might be differentially regulated during different stages of embryonic muscle development (Fig. 5C) [37]. These overlapping genes were significantly enriched in muscle-related terms, such as skeletal muscle tissue development, skeletal muscle organ development and actin cytoskeleton organization (Additional file 3: Table S3). Only one pathway, namely Rap1 signaling pathway, showed significant enrichment (Additional file 4: Table S3).

**Fig. 6:**
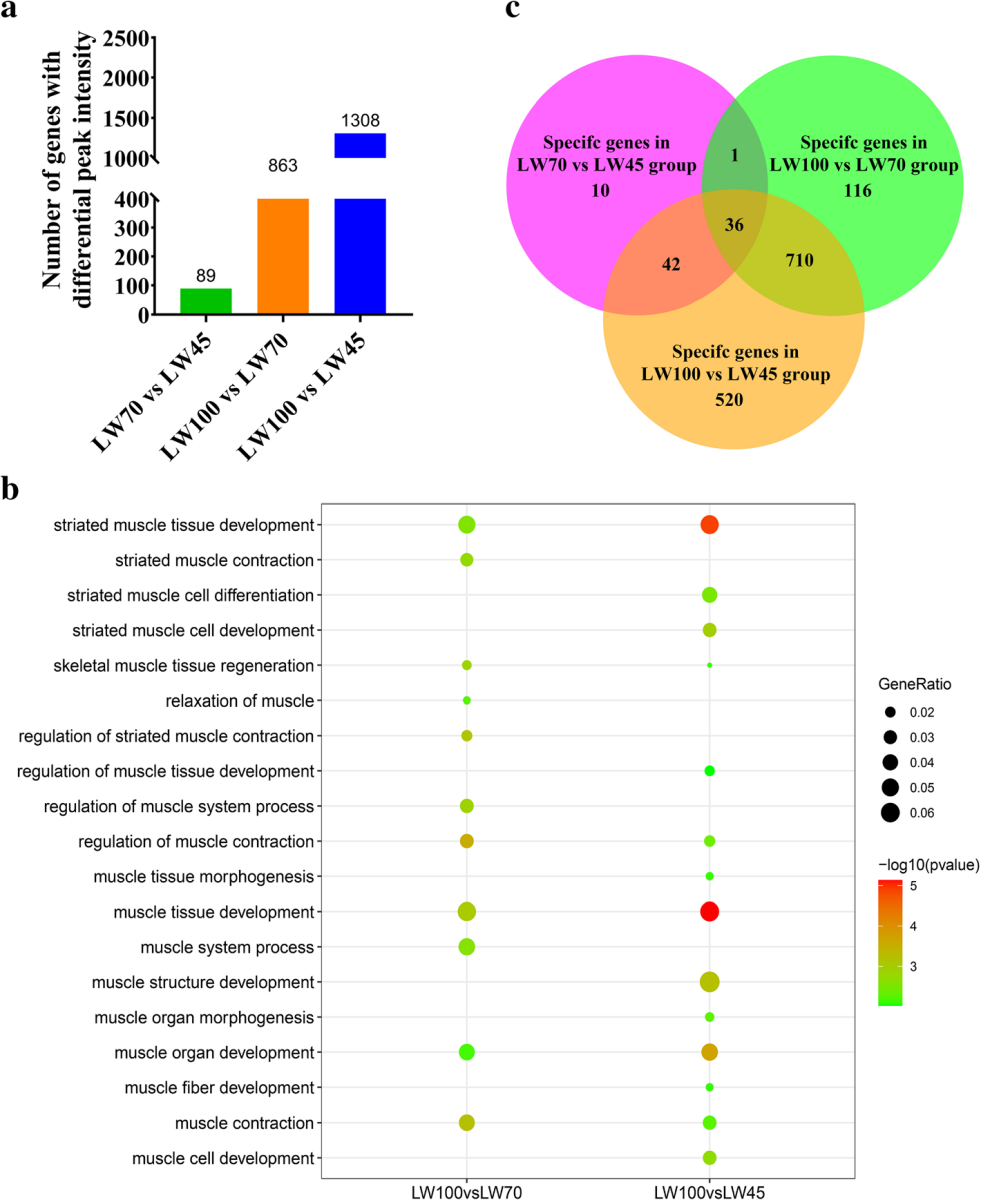
Functional analysis of genes associated with DPIs. **a** Numbers of genes with DPIs in ACRs shared among stages. **b** Some of muscle related BPs in the LW100 vs LW70 and LW100 vs LW45 groups. The dot color represents the statistical significance level of enrichment (*P* value); the dot size indicates the proportion of genes enriched for the corresponding terms. **c** Numbers of shared and stage-specific genes associated with DPIs identified from the LW70 vs LW45, LW100 vs LW70 and LW100 vs LW45 comparisons

For the analysis of common peaks, we first annotated the above-mentioned DPI to the genome, and then performed functional analysis using the annotated genes. A total of 88, 863 and 1308 genes were obtained from each comparison (Fig. 6a). Similar to the results obtained for genes with stage-specific ACRs, the genes annotated by DPIs in each comparison with the exception of the LW70 vs LW45 comparison were significantly enriched in many muscle-related related BPs (Additional file 5: Table S4 and Fig. 6b), such as muscle organ development, muscle tissue development and striated muscle tissue development. Nine terms were obtained for LW70 vs LW45 comparison and most of these were significantly associated with cardiac-related development (Additional file 4: Table S4). Several metabolism-related pathways, such as fatty acid metabolism, which can provide energy to muscles for exercise, were significantly enriched in the genes showing DPIs between the LW70 vs LW45 comparison [38]. Moreover, some custom developmental pathways that have been proven to play an essential role in muscle development, such as the Wnt signaling pathway and TGF-beta signaling pathway were significantly enriched in the genes identified from the LW100 vs LW70 and LW100 vs LW45 comparisons.

In addition, among the genes with DPIs, we found 36 genes were identified by all three comparisons (Fig. 6c). And those were significantly enriched in 11 terms. Moreover, most of those were found to be related to fundamental cell functions (Additional file 5: Table S4).

### Identification of regulatory DNA elements at different stages during embryonic development

**Fig. 7:**
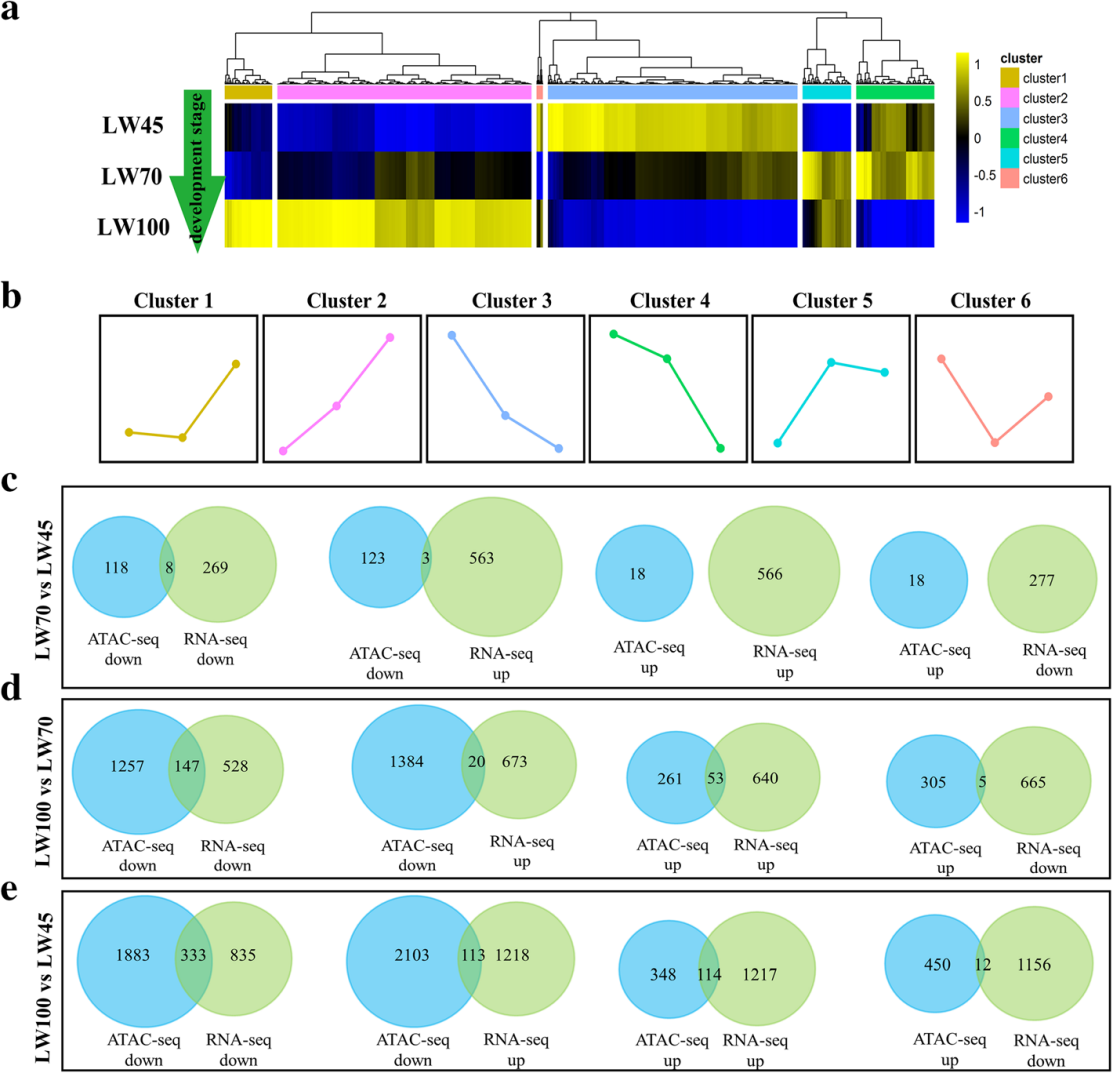
Identification of regulatory DNA elements at different stages. **a** Heatmap of the expression levels of DEGs at different developmental stages. **b** Expression profiles of all DEGs in six clusters. **c-e** Overlap of DPI-related genes identified ATAC-seq with the DEGs detected by RNA-seq. ATAC-seq down: DPI-related genes were downregulated in ATAC-seq; ATAC-seq up: DPI-related genes were upregulated in ATAC-seq; RNA-seq down: DEGs were downregulated in RNA-seq; RNA-seq up: DEGs were upregulated RNA-seq

The expression levels of the genes showed substantial difference at different stages during embryonic muscle development. We identified 2749 DEGs through pairwise differential expression analysis among the different stages. All of these DEGs could be divided into six clusters based on their expression patterns (Fig. 7a). The expression level of genes in cluster 3 and 4 decreased gradually from LW45 to LW100, whereas the genes in cluster 2 presented expression trends opposite to those of the genes in cluster 3 and 4. The genes in cluster 5 were highly expressed at the LW70 stage but displayed sharp changes at the LW45 stage. The genes in cluster 1 were expressed at low levels at the LW45 and LW70 stages but showed at sharp increase in expression at the LW100 stage. The genes in cluster 6 exhibited their highest expression at LW45 and their lowest expression at LW70 (Fig. 7b).

We then integrated the genes with stage-specific peaks in their proximal promoter regions with the DEGs revealed by RNA-seq and found that the genes containing stage-specific ACRs at LW45 showed the most enrichment in cluster 3, but this enrichment was not significant (Table 2). In addition, genes containing stage-specific ACRs at LW70 were significantly enriched in clusters 3 and 4 (Table 2), and genes with LW100 stage-specific ACRs were significantly enriched in clusters 2 and 5, which included the genes with the high expression levels at LW100 (Table 2). These findings suggested that the alterations in gene expression might be related to the regulation of ACRs.

**Table 2:** Significance of the overlap between specific ACR-related genes and DEGs in six clusters

	Gene associated with specific ACRs in LW45	Gene associated with specific ACRs in LW70	Gene associated with specific ACRs in LW100
Cluster1	1	1	0.31
Cluster2	1	1	5.96 E-04
Cluster3	0.06	0.05	1
Cluster4	0.07	0.04	0.96
Cluster5	1	0.71	3.60 E-02
Cluster6	1	1	0.48

We further investigated the CREs within ACRs at proximal promoter in clusters 2, 3 and 5 using HOMER. Several known motifs associated with muscle development were significantly enriched at all stages (Additional file 2: Fig. S6- S9). For example, MyoG, which is a well-known fundamental regulator of the skeletal muscle lineage during the embryonic period, was significantly enriched at the LW45-specific ACRs in cluster 3 genes (Additional file 2: Fig. S6) [39]. In addition, muscle development-related TFs, such as Mef2d, Mef2a and Mef2c, which are members of the MEF2 family of TFs, were significantly identified at LW100-specific ACRs in cluster 2 genes (Additional file 2: Fig. S8-S9) [40]. These results suggest that the identified TFs regulate a considerable proportion of genes and might play important roles during embryonic muscle development. Notably, a potential transcriptional repressor that plays important roles in cell cycle regulation, E2F6, was detected at the LW70-specific ACRs in cluster 3 genes, which exhibited peak expression at LW45 and showed sharp decrease in expression at the following stages (Additional file 2: Fig. S7) [41, 42]. We hypothesize that the presence of this transcriptional repressor might decrease the expression levels of some of the genes in cluster 3 at the LW70 stage. These results show that the regulation of genes by TFs is dynamic during embryonic muscle development.

For further analysis of genes with common peaks, we integrated the DPI-related genes identified ATAC-seq with the DEGs detected by RNA-seq (Fig. 7c-e). A total of 8, 147 and 333 downregulated genes were identified from the LW70 vs LW45, LW100 vs LW70 and LW100 vs LW45 comparisons, respectively. Whereas 0, 53 and 114 upregulated genes were found from the LW70 vs LW45, LW100 vs LW70 and LW100 vs LW45 comparisons, respectively. This result suggests that ACRs might play important roles in regulating the expression of genes.

To further explore the functions of these genes in detail, GO and KEGG pathway analyses were performed (Additional file 6: Table S5). The results obtained with the LW100 vs LW70 and LW100 vs LW45 comparisons showed the enrichment of several muscle-related terms, such as muscle contraction, regulation of muscle system process and regulation of muscle contraction. The significantly enriched GO terms obtained for LW70 vs LW45 comparison were mainly associated with synaptic-related functions and no significantly enriched pathways were identified with this comparison. The most significantly enriched pathway, namely, the calcium signaling pathway, was identified from both the LW100 vs LW70 and LW100 vs LW45 comparisons.

In addition, for the LW70 vs LW45, LW100 vs LW70 and LW100 vs LW45 comparisons, three, 20 and 113 downregulated ACR-related genes and no, fibe and 12 upregulated ACR--related genes indenrified by ATAC-seq were found to be upregulated and downregulated based on the RNA-seq data, respectively (Fig. 7c-e). We sought to identify whether these differentially altered ACRs showed enrichment for a particular transcriptional repression factor. Ultimately, some known transcriptional repression factors were identified using HOMER, except in the LW70 vs LW45 comparison (Additional file 2: Fig. S10-S12). For example, the TF OTX2, was found to be enriched in the LW100 vs LW45 comparison ((Additional file 2: Fig. S12). In addition, CTCF was identified in both the LW100 vs LW70 and LW100 vs LW45 comparisons, which suggests that some of the genes identified in these two comparisons might be regulated by the same TFs during embryonic muscle development (Additional file 2: Fig. S11-S12).

## Discussion

The development of skeletal muscle during the embryonic period determines muscle growth [43, 44]. Pigs undergo primary and secondary fiber formation during embryonic muscle development via a series of complex regulatory mechanisms [7]. The genome-wide chromatin accessibility affects BPs at different developmental stages, including embryonic muscle development, by regulating TF activity. In the present study, we analyzed the chromatin accessibility and transcriptomes of *longissimus dorsi* from LW pig embryos (at 45, 70, 100 dpc). Due to the result of ATAC-seq and RNA-seq could be influenced not only by the development stages but also the genetics of the animals, so the donor sows at LW45, LW70 and LW100 stages in this study are full-sibs in order to minimize the difference in genetic background. To our knowledge, this study constitutes the first systematic investigation of chromatin accessibility in LW pig embryonic skeletal muscle by ATAC-seq and conjunction with transcriptomic analysis.

ATAC-seq has rapidly become the preferred approach for the study of chromatin accessibility due to its simplicity, e.g., shorter experimental time and need for fewer materials. However, its application still has several limitations. For example, this method is performed based on the activity of Tn5 transposase which exhibits a tiny preference for a specific DNA sequence [45, 46]. The limitation or inappropriateness of generalized methods might challenge the analysis of ATAC-seq data [15]. Moreover, it is becoming increasingly appreciated that the interactions between proteins and DNA are highly dynamic, and thus, the current methods for profiling chromatin accessibility might not be capable to disclose these interactions. As a result, the particular region obtained in this study is the relatively stable open part of chromatin [47].

The crucial ATAC-seq technique involves library construction using the hyperactive transposase Tn5. Therefore, we analyzed the insert size distribution and peak signal enrichment and observed a clear pattern in the fragment distribution: the nucleosome-depleted and mononucleosomal spanning regions accounted for half of the total reads. In addition, the ACRs were concentrated at the proximal TSSs, which is consistent with the fact that chromatin around TSSs throughout the genome is more accessible than that in surrounding genomic regions. Overall, our results are in excellent agreement with those of many studies [48, 49]. Our genome-wide identification of peaks in muscle tissue from embryos at different stages by ATAC-seq revealed that the number of peaks increased with increases in the age of the embryo, which indicats that chromatin accessibility is involved in the regulation of embryonic muscle development. During the period of secondary fiber proliferation and differentiation (70–100 dpc), chromatin accessibility in LW pigs increased significantly, which indicats that the regulatory mechanisms of the proliferation and differentiation of secondary fibers might be more complicated than those of primary fibers.

The chromatin accessibility landscape has been profiled in many species, such as humans, mice and plants [49–51], and the resulting data have provided references for deep genome research. Based on an early theory, the hierarchical compaction and organization of nucleosomes in eukaryotes can divide the genome into inactive regions and active regions, such as promoters and enhancers, that participate in subsequent transcription processes [52]. Based on this theory, the location of ACRs has been found to be enriched in promoters in many studies, including the present study [53, 54]. Consistent with our results, the majority of peaks have been mapped to intergenic regions and introns, followed by promoters and exons in approximately equal proportions, and the distributions of peaks in the genome show similarities among different species despite differences in their genome sizes and degrees of genome annotation [19, 51, 55, 56]. We speculate that this similarity might indicate that the distribution of open chromatin among various species or tissues exhibits little conservation.

In our study, the ACR number and ACR length, which were associated with the gene expression levels, showed variation among the different stages tested. We found that ACRs in proximal promoter regions were significantly longer than those in other regions, whereas those in intergenic regions were the shortest. Moreover, the genes with longer ACRs or multiple ACRs in their proximal promoter regions tended to exhibit higher expression levels. These observations suggest that the chromatin accessibility of promoters can alter the expression levels of associated genes. Similar results have been found in many previous studies, which can verify our results to some extent [57]. These findings might indicate a conserved regulatory pattern for gene expression mediated by chromatin accessibility among different species.

ACRs show substantial variation among different tissues and cell types during the developmental stage [54, 58, 59]. For example, widespread decreases in chromatin accessibility have been demonstrated to occur in age-related macular degeneration, and a few common ACRs have been found to be shared among different tissues [60]. In the current study, both specific and shared ACRs were uncovered during embryonic muscle development. Genes associated with stage-specific or common ACRs were enriched in a series of classic muscle signaling pathways and various muscle-related GO terms. Notably, several genes harbored dynamic ACRs or exhibited DPIs at different stages. These results show that ATAC-seq can be used to effectively analyze the effects of epigenetics on muscle development.

Moreover, through a combined analysis of chromatin accessibility and transcriptome data, we further investigated the interactions between TFs and ACRs and analyzed the reasons for the specific spatiotemporal expression of genes during embryonic muscle development in pigs. In our study, the ATAC-seq and RNA-seq data revealved contradictory trends in expression for some genes annotated with DPIs. Hence, we speculate that some potential transcriptional repressors might play important roles in embryonic muscle development.

Through motif analysis, we identified an enriched TF from the LW100 vs LW45 comparison, OXT2, which is a potential transcriptional repressor. OTX2, a TF involved in brain development, can repress MyoD1 expression by binding its homeobox domain to the MyoD1 core enhancer to suppress muscle differentiation [61]. Moreover, another key TF, CTCF, was identified from both the LW100 vs LW70 and LW100 vs LW45 comparisons. CTCF, which is a highly conserved zinc-finger DNA-binding protein, has been found to play a role as a transcriptional repressor of the *Myc* gene and to be involved in the occurrence of various cardiovascular diseases [62, 63]. CTCF has a very large number of binding sites in the mammalian genome (40,000–80,000), and these are mainly concentrated in intergenic regions and introns and overlap with enhancer and promoter sequences. According to previous studies, the binding of CTCF to promoter or enhancer regions often exerts an inhibitory effect [62]. Therefore, we speculate that the inconsistent trend obtained with ATAC-seq and RNA-seq might fue to the binding of CTCF to the promoter or enhancer regions of some genes.

## Conclusions

Taken together, the findings of this study demonstrate the dynamic changes in chromatin accessibility that occurs during embryonic muscle development. An integrated analysis of ATAC-seq and RNA-seq data showed that the modulation of ACRs can significantly alter the associated genes and identified numerous valuable CREs and potential transcriptional repressors involved in the regulation of muscle development. This study can therefore be used as a reference for future research on muscle development in mammals.

## Supplementary Information

**Additional file 1: Table S1.** ATAC-seq sequencing statistics of the samples

**Additional file 2: Fig. S1.** Insertion size distribution of all libraries. A, B and C show the insertion sizes of the LW45, LW70 and LW100 libraries, respectively. **Fig. S2.** Heatmap of the peak signals across the gene body in each library. A, B and C show the results for the LW45, LW70 and LW100 libraries, respectively. **Fig. S3**. Heatmap of all genes at all stages. **Fig. S4.** Percentages of ACR-associated gene expression levels in different groups. The genes contained multiple ACRs in the proximal promoter regions were grouped according to the total peak length and were then divided into three equal groups (the top, middle and bottom groups). The expression levels of the genes were divided into five groups based on the FPKM values: 0–2, 2–5, 5–10, 10–30, and 30-more. **Fig. S5.** The landscape of stage-specific ACRs related genes and DPIs related genes. **(a)** Heatmap of all expressed genes contained stage-specific ACRs at the LW45, LW70 and LW100 stages. The red color indicates high expression level; whereas the green color indicates low expression level. **(b)** The distribution of the pearson correlation coefficient between the peak intensity and expression level of each DPI-related gene. **Fig. S6.** Enrichment of known TF motifs identified in the proximal promoter regions of cluster 3 genes with specific ACRs at the LW45 stage. **Fig. S7**. Enrichment of known TF motifs identified in the proximal promoter regions of cluster 3 genes with specific ACRs at the LW70 stage. **Fig. S8**. Enrichment of known TF motifs identified in the proximal promoter regions of cluster 2 genes with specific ACRs at the LW100 stage. **Fig. S9**. Enrichment of known TF motifs identified in the proximal promoter regions of cluster 5 genes with specific ACRs at the LW100 stage. **Fig. S10**. Enrichment of known TF motifs identified from the common ACRs showing DPIs in the LW70 vs LW45 comparison. **Fig. S11**. Enrichment of known TF motifs identified from the common ACRs showing DPIs in the LW100 vs LW70 comparison. **Fig. S12**. Enrichment of known TF motifs identified from the common ACRs showing DPIs in the LW100 vs LW45 comparison.

**Additional file 3: Table S2**. Information on the RNA-seq libraries.

**Additional file 4: Table S3.** GO and KEGG pathway analyses of stage-specific ACRs among different stages. LW45_GO_terms, LW70_GO_terms and LW100_GO_terms: results from the GO analyses of stage-specific ACRs at the LW45, LW70 and LW100 stages, respectively; LW45_pathways, LW70_pathways and LW100_pathways: results from the KEGG pathway analyses of stage-specific ACRs at the LW45, LW70 and LW100 stages; common_GO_terms: results from the GO analyses of common genes with stage-specific ACRs overlapped among the three stages. common_pathways: results from the KEGG pathway analyses of common genes with stage-specific ACRs overlapped among the three stages.

**Additional file 5: Table S4.** GO and KEGG pathway analyses of common ACRs with DPIs. LW70vsLW45_GO, LW100vsLW70_GO and LW100vsLW45_GO: results from the GO analyses of common ACRs with DPIs identified from the LW70 vs LW45, LW100 vs LW70 and LW100 vs LW45 comparisons, respectively; LW70vsLW45_pathways, LW100vsLW70_pathways and LW100vsLW45_pathways: results from the KEGG pathway analyses of common ACRs with DPIs identified from the LW70 vs LW45, LW100 vs LW70 and LW100 vs LW45 comparisons, respectively. common_GO_terms: results from the GO analyses of common genes among three comparisons.

**Additional file 6: Table S5.** GO and KEGG pathway analyses of genes that were identified as both DPI-related genes and DEGs. LW70vsLW45_GO, LW100vsLW70_GO and LW100vsLW45_GO: results from GO analyses of overlapping genes identified from the LW70 vs LW45, LW100 vs LW70 and LW100 vs LW45 comparisons, respectively; LW100vsLW70_pathways and LW100vsLW45_pathways: results from the KEGG pathway analyses of overlapping genes identified from the LW100 vs LW70 and LW100 vs LW45 comparisons, respectively.

## Data Availability

The sequencing datasets supporting the conclusions of this article are available in the BIG Data Center (http://bigd.big.ac.cn/) with the accession code CRA003275.

## Abbreviations

dpc: Days post-coitus
TF: Transcription factors
QTL: Quantitative trait locus
DNase-seq: Deoxyribonuclease I (DNase I)-hypersensitive Site sequencing
ATAC-seq: Transposase-accessible chromatin with high-through sequencing
FAIRE-seq: Formaldehyde-assisted isolation of regulatory elements sequencing
D/LW: Large White
ACR: Accessible chromatin regions
TSS: Transcription start sites
TTS: Transcriptional termination sites
BP: Biological processes
DPI: Differential peak intensity
DEGs: Differential expressed genes
