# Supplementary material for: The landscape of chromatin accessibility in skeletal muscle during embryonic development in pigs
Source: J Anim Sci Biotechnol. 2021 May 3;12:56. doi: 10.1186/s40104-021-00577-z (PMC8091695; doi:10.1186/s40104-021-00577-z)

Fig. S1.


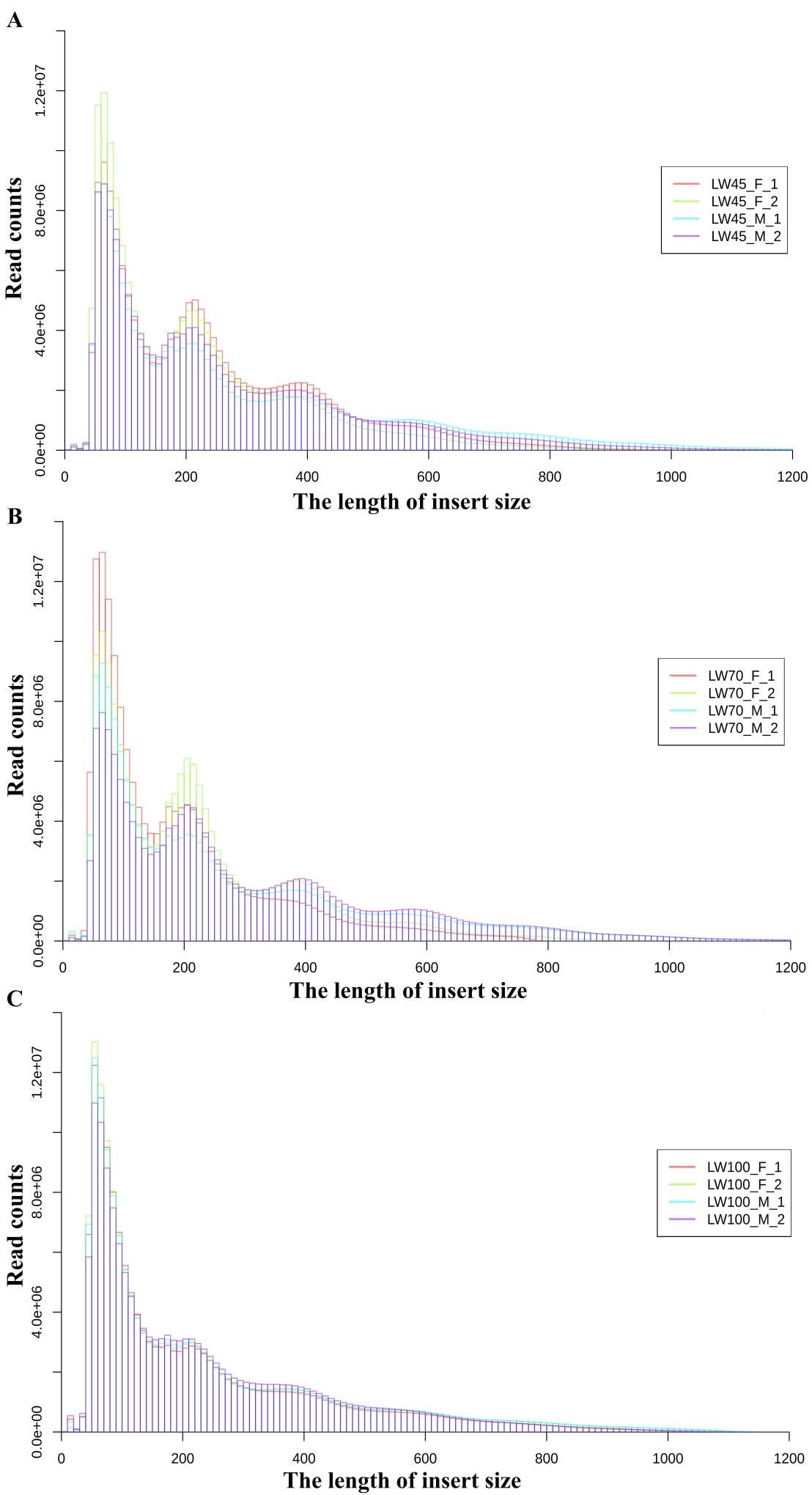


Fig. S2.


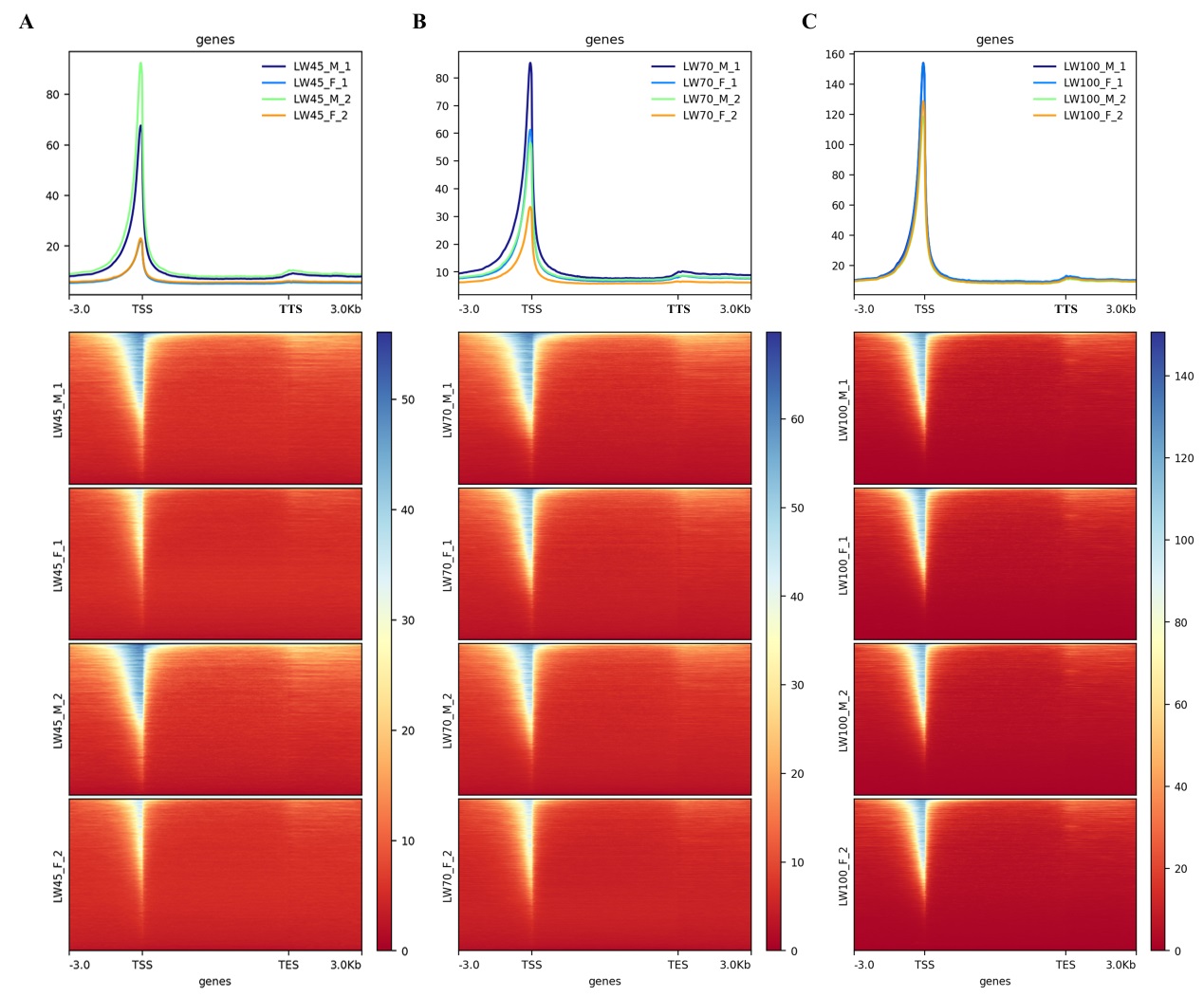


Fig. S3.


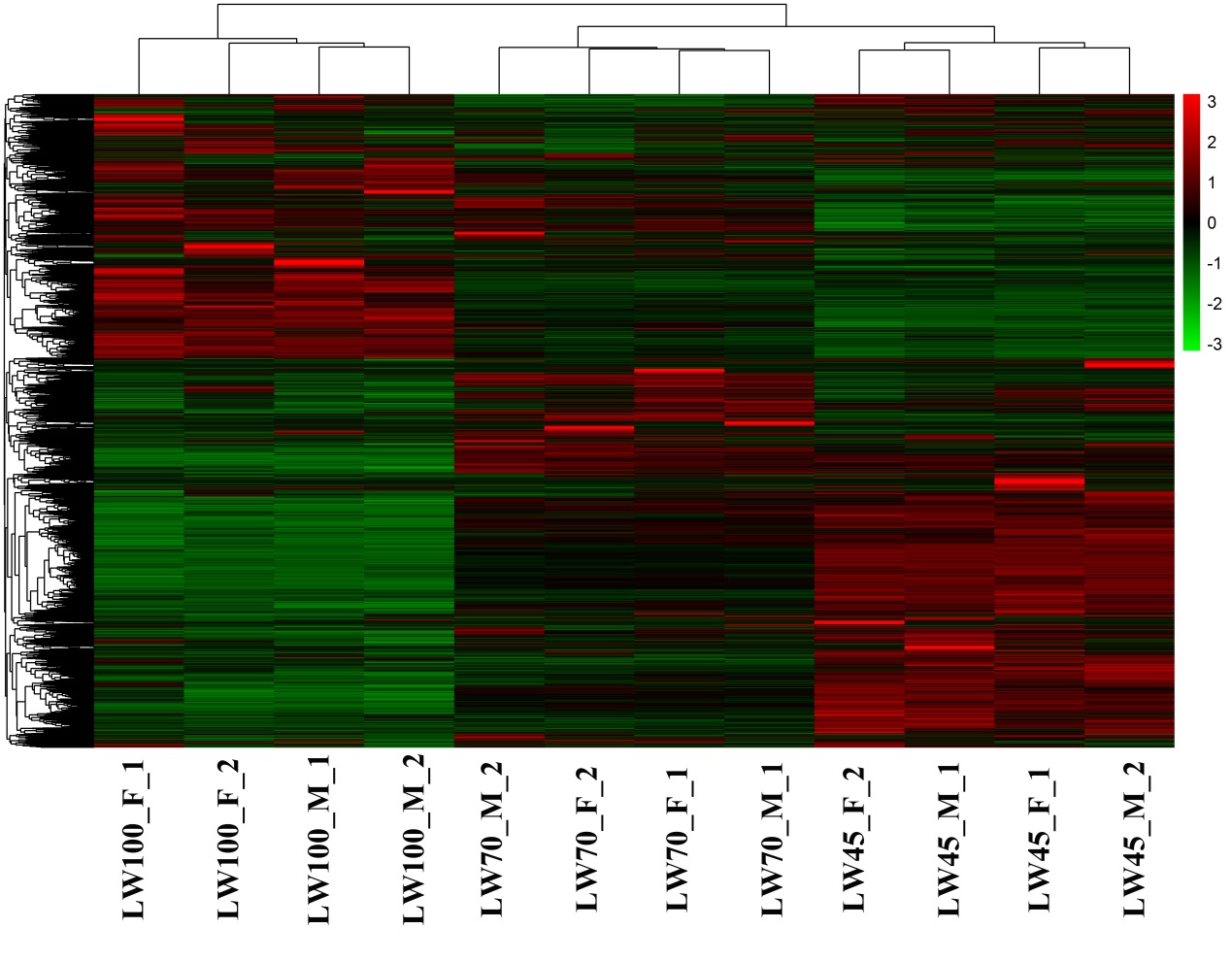


Fig. S4


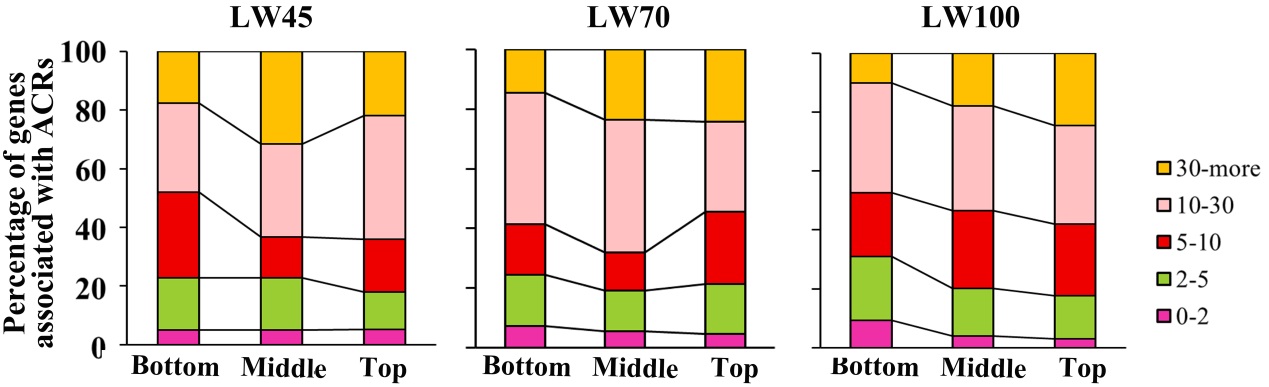


Fig. S5


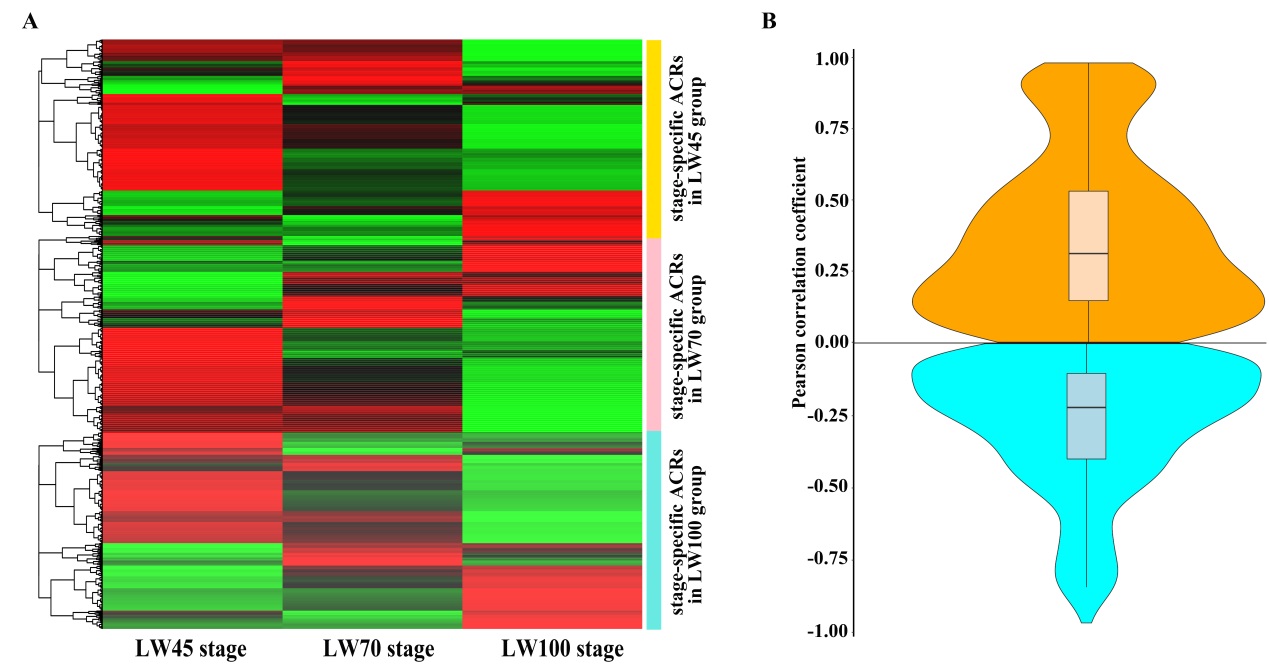


Fig. S6.


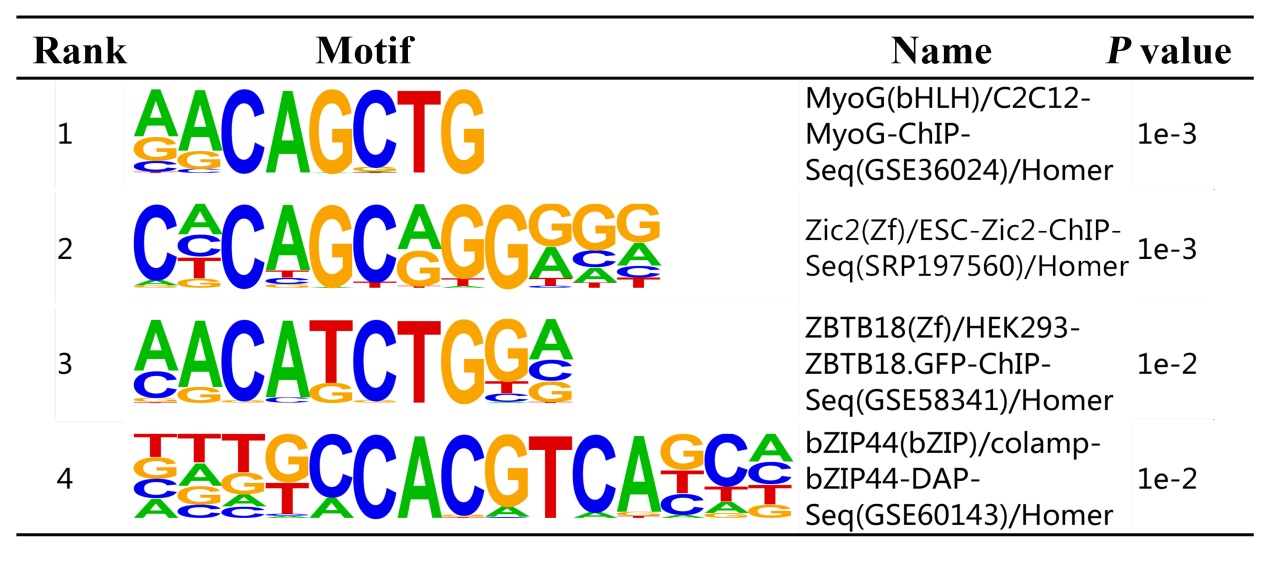


Fig. S7.


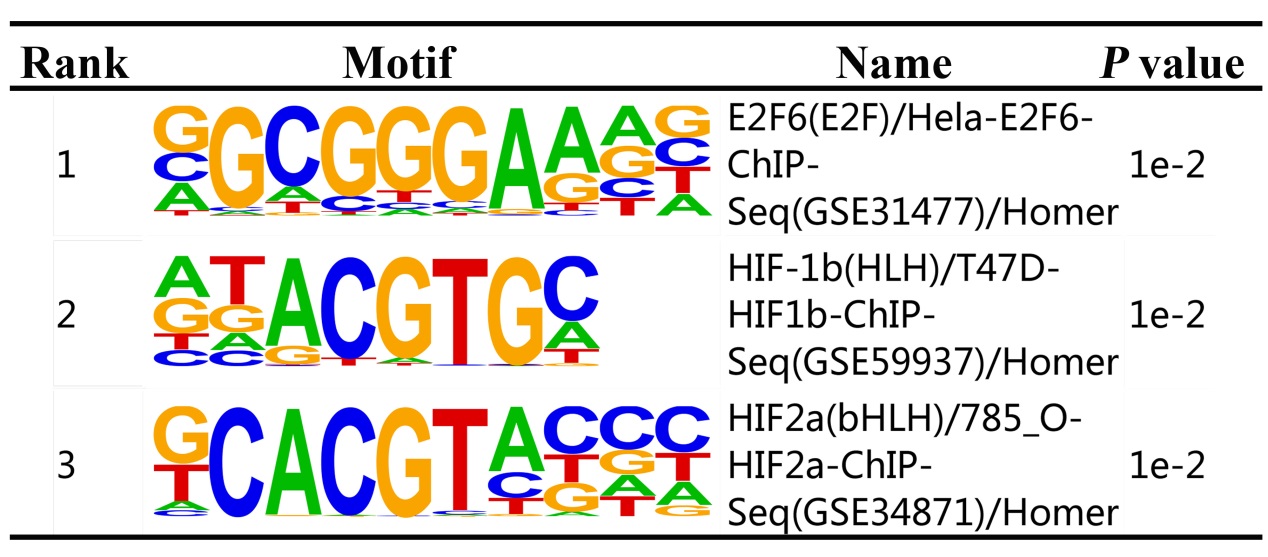


Fig. S8


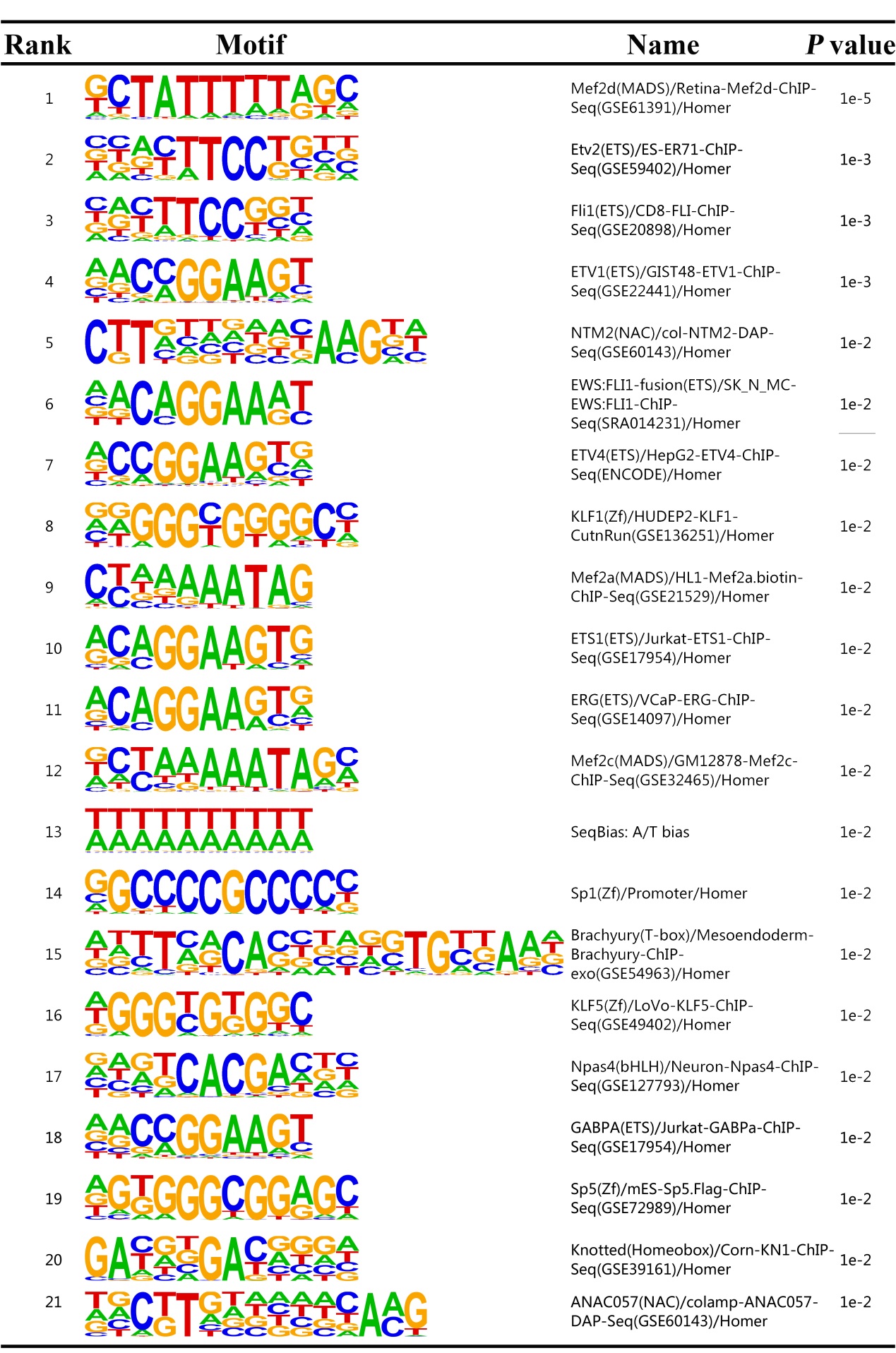


(Legend continued on next page)


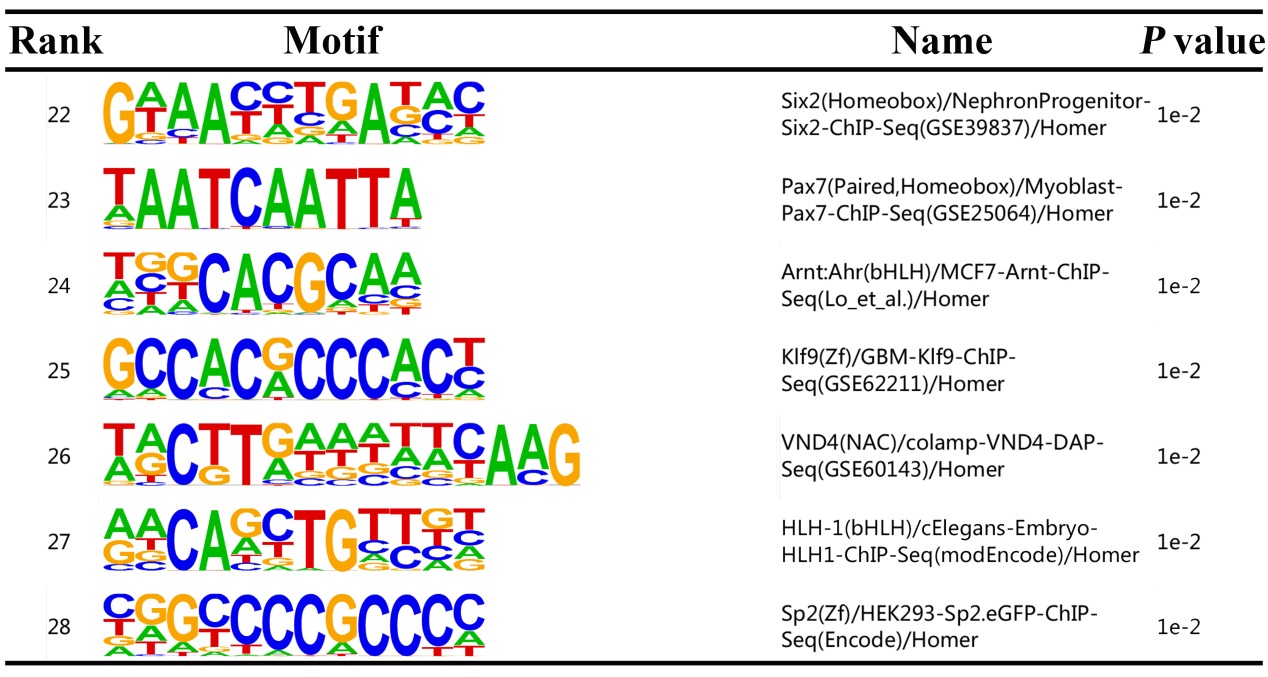


Fig. S9.


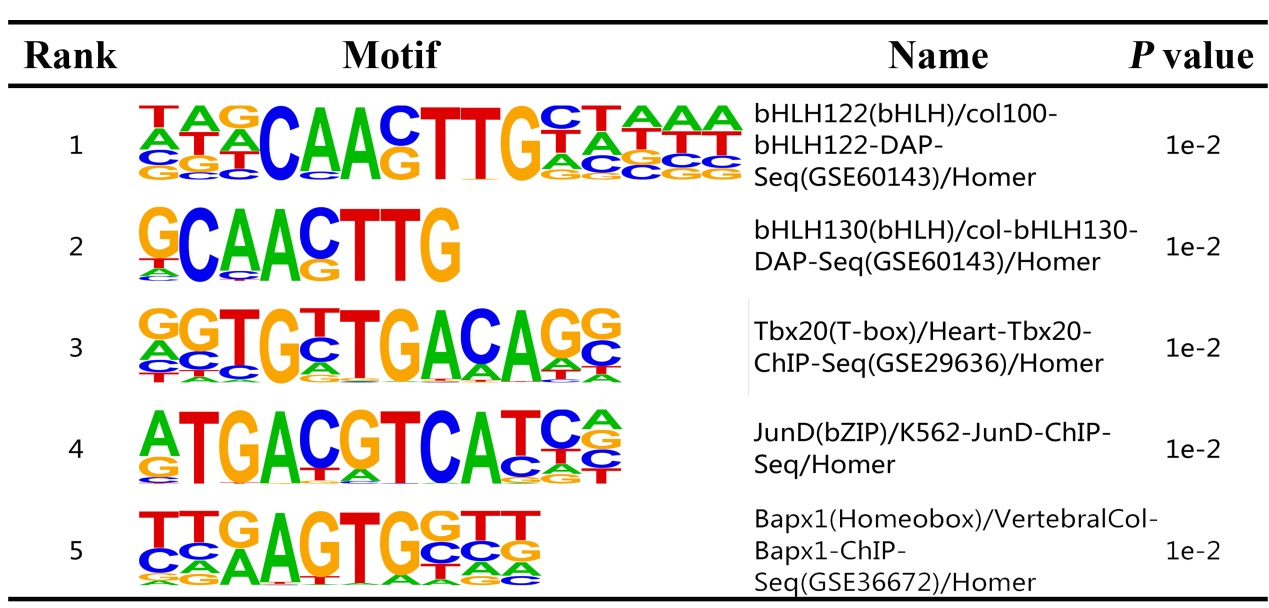


Fig. S10.


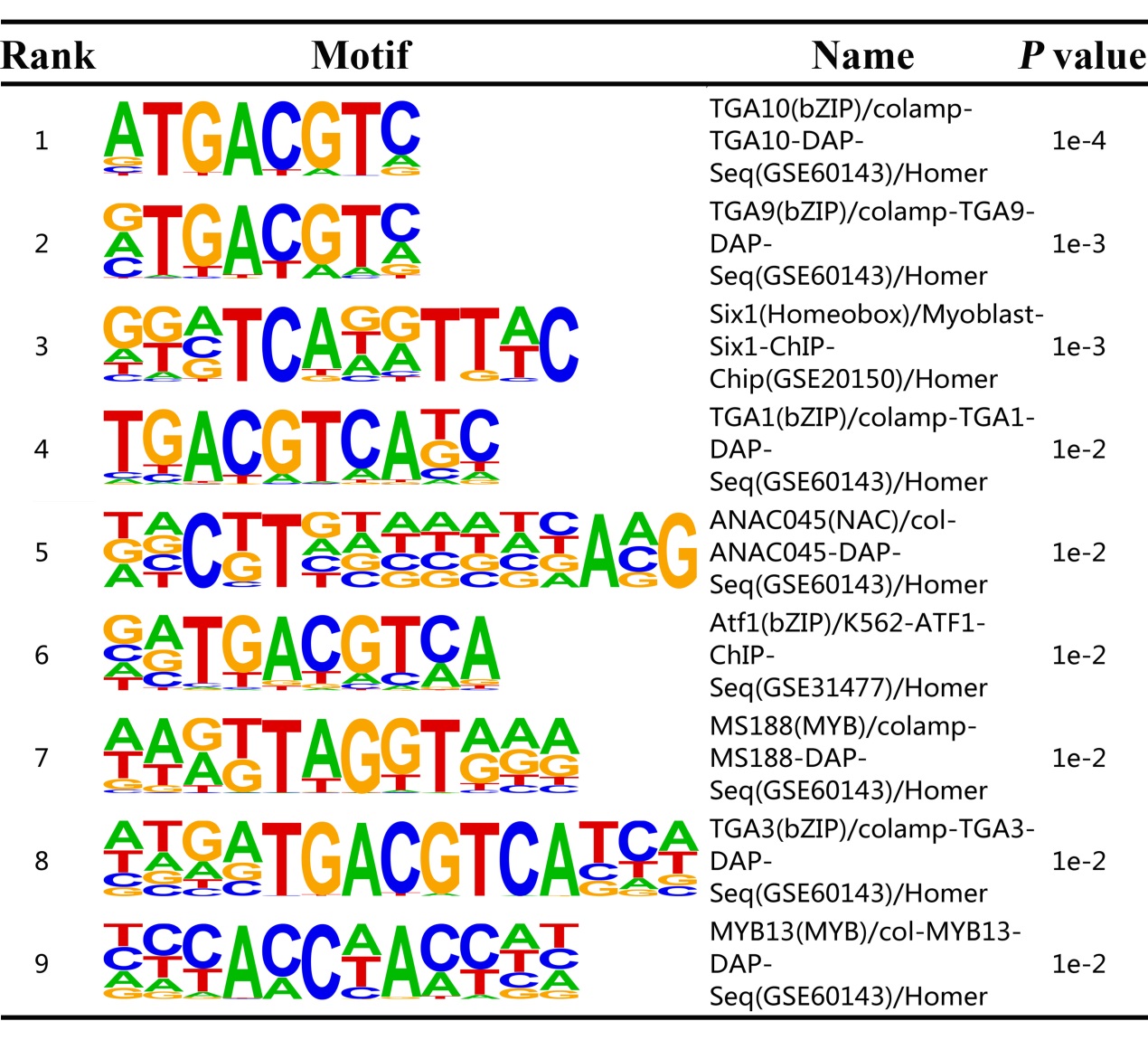


Fig. S11.


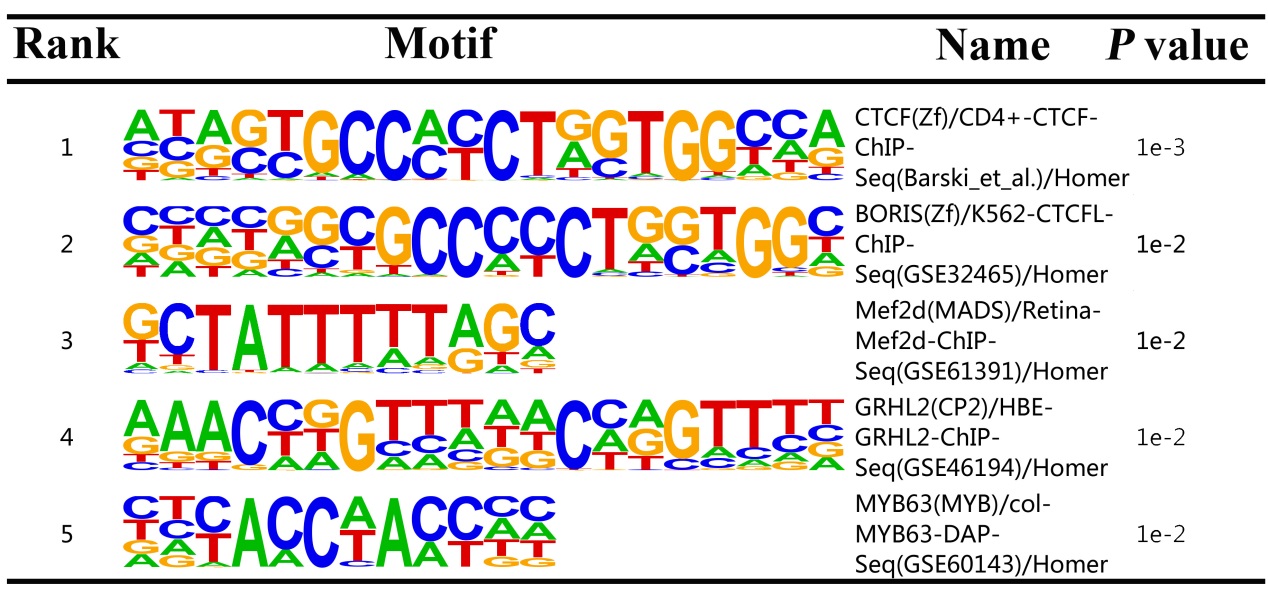


Fig. S12.


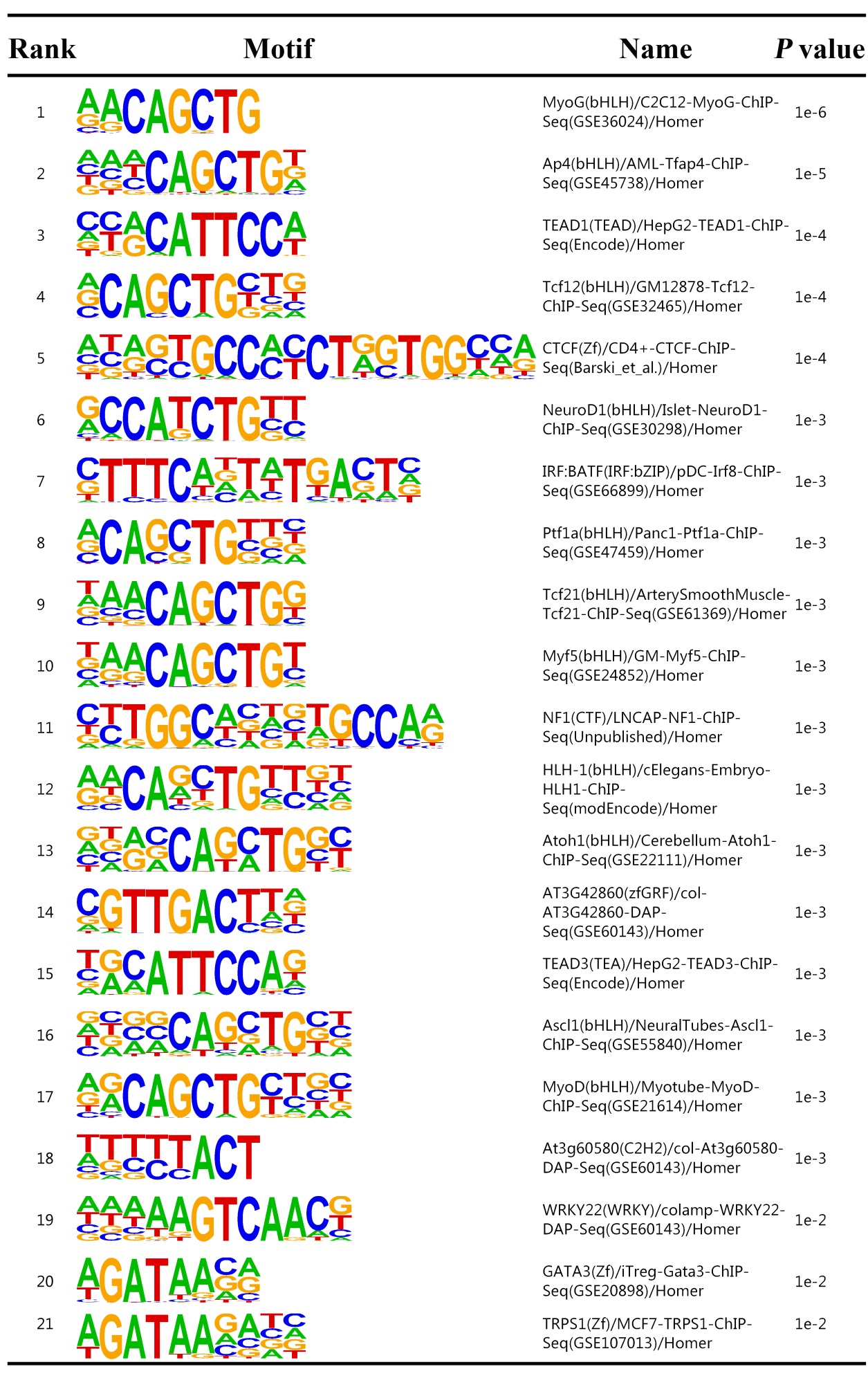
 (Legend continued on next page)


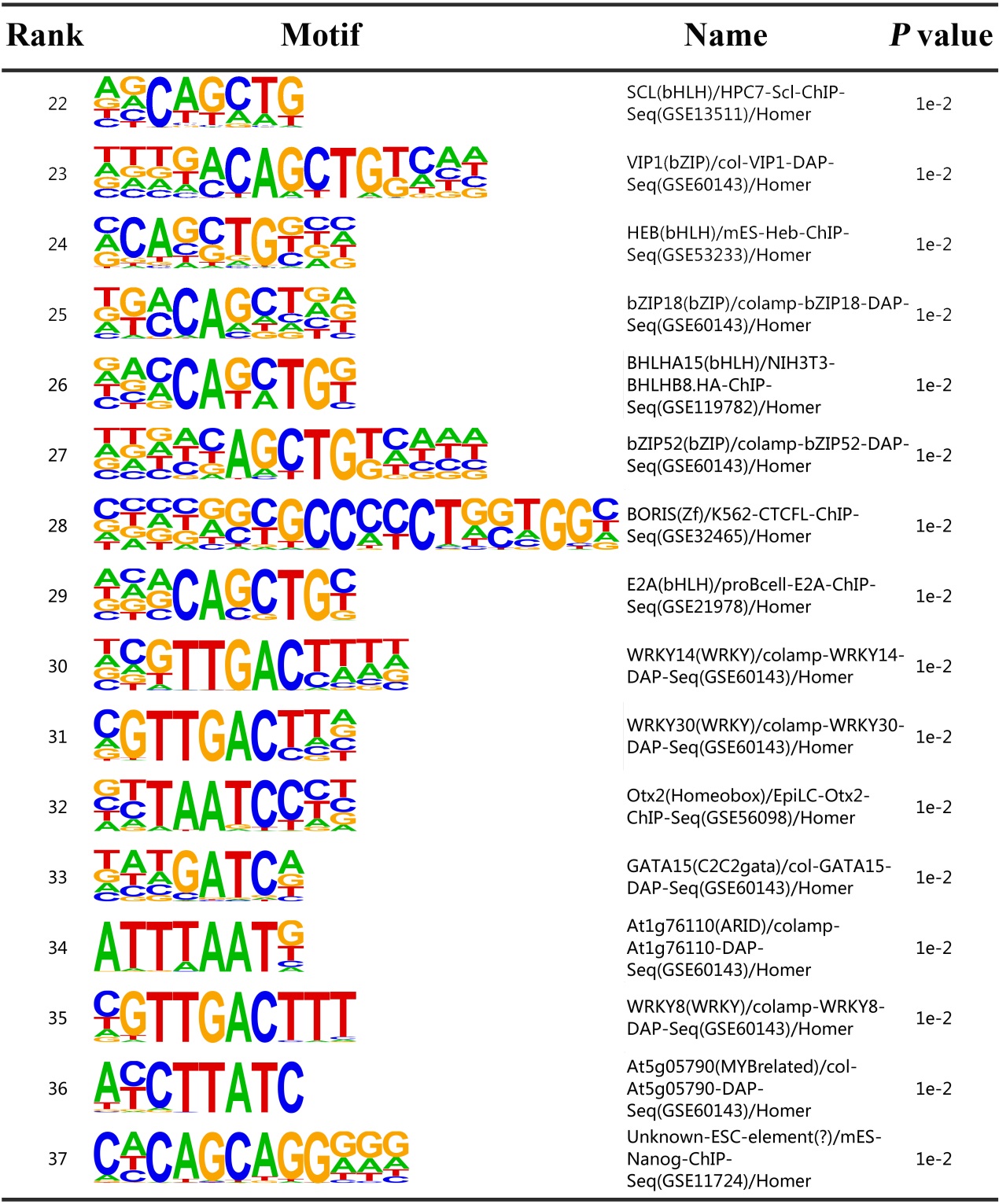

Supplement: Supplementary file 2 — Additional file 2: Fig. S1. Insertion size distribution of all libraries. A, B and C show the insertion sizes of the LW45, LW70 and LW100 libraries, respectively. Fig. S2. Heatmap of the peak signals across the gene body in each library. A, B and C show the results for the LW45, LW70 and LW100 libraries, respectively. Fig. S3. Heatmap of all genes at all stages. Fig. S4. Percentages of ACR-associated gene expression levels in different groups. The genes contained multiple ACRs in the proximal promoter regions were grouped according to the total peak length and were then divided into three equal groups (the top, middle and bottom groups). The expression levels of the genes were divided into five groups based on the FPKM values: 0–2, 2–5, 5–10, 10–30, and 30-more. Fig. S5. The landscape of stage-specific ACRs related genes and DPIs related genes. (a) Heatmap of all expressed genes contained stage-specific ACRs at the LW45, LW70 and LW100 stages. The red color indicates high expression level; whereas the green color indicates low expression level. (b) The distribution of the pearson correlation coefficient between the peak intensity and expression level of each DPI-related gene. Fig. S6. Enrichment of known TF motifs identified in the proximal promoter regions of cluster 3 genes with specific ACRs at the LW45 stage. Fig. S7. Enrichment of known TF motifs identified in the proximal promoter regions of cluster 3 genes with specific ACRs at the LW70 stage. Fig. S8. Enrichment of known TF motifs identified in the proximal promoter regions of cluster 2 genes with specific ACRs at the LW100 stage. Fig. S9. Enrichment of known TF motifs identified in the proximal promoter regions of cluster 5 genes with specific ACRs at the LW100 stage. Fig. S10. Enrichment of known TF motifs identified from the common ACRs showing DPIs in the LW70 vs LW45 comparison. Fig. S11. Enrichment of known TF motifs identified from the common ACRs showing DPIs in the LW100 vs LW70 comparison. Fig. S12 [file 40104_2021_577_MOESM2_ESM.docx]
